# The method simulating spontaneous pain in patients with nociplastic pain using rats with fibromyalgia-like condition

**DOI:** 10.1016/j.mex.2020.100826

**Published:** 2020-02-26

**Authors:** Shigeharu Tanei, Machiko Miwa, Miku Yoshida, Reina Miura, Yukinori Nagakura

**Affiliations:** a Faculty of Pharmaceutical Sciences, Aomori University, 2-3-1 Kohbata, Aomori 030-0943 Japan; b Faculty of Pharmaceutical Sciences, Nihon Pharmaceutical University, 10281 Komuro, Ina-machi, Kitaadachi-gun, Saitama 362-0806, Japan; c School of Pharmacy, International University of Health and Welfare, 2600-1 Kitakanemaru, Ohtawara, Tochigi 324-8501, Japan; d School of Pharmacy in Fukuoka, International University of Health and Welfare, 137-1 Enokizu, Okawa-city, Fukuoka 831-8501, Japan

**Keywords:** Nociplastic pain, Spontaneous pain, Fibromyalgia-like rat, Grimace scale, Long-lasting pain, Efficacy translation

## Abstract

The method shown in this article simulates spontaneous pain in patients with nociplastic pain using rats; the measurement with this method could be related to better translation of analgesic efficacies of therapeutic compounds between rats and humans. Nociplastic pain occurs in various disorders including fibromyalgia. Because the pain in patients occurs without an external stimulus, we assessed spontaneous pain in rats. The grimace scale, a methodology for rating facial expression, has been used for measuring spontaneous pain in animals. However, the responses in animals have been rather short-lived, and the scale has never been applied to animals exhibiting nociplastic pain. Here, we apply the rat grimace scale (RGS) to the reserpine-induced fibromyalgia-like rat, which induces nociplastic pain. The ratings of the orbital tightening, nose/cheek flattening, and changes in characteristics of ears and whiskers by three raters, who were blinded to the treatment allocated to rats, demonstrated substantial, long-lasting change in facial expression of rats. In this article, reference images for raters, and sample images used for rater training are provided. All raters independently indicated that the RGS score is significantly elevated with this methodology in reserpine-induced fibromyalgia-like rats.

• The grimace scale, a method for rating facial expression, is applied to the reserpine-induced fibromyalgia-like rat, which manifests nociplastic pain.
• Facial expression change in the reserpine-induced fibromyalgia-like rat is substantial and long-lasting.
• Elevation of the RGS score in the reserpine-induced fibromyalgia-like rat may simulate spontaneous pain in patients with nociplastic pain.

## Graphical abstract

**Figure:**
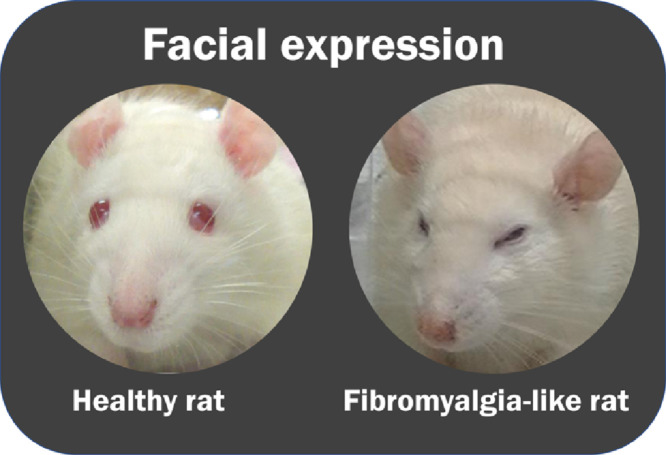

**Table:** Specification Table

Subject Area:	Pharmacology, Toxicology and Pharmaceutical Science
More specific subject area:	Pain and analgesia
Method name:	Facial expression score in animals exhibiting nociplastic spontaneous pain
Name and reference of original method:	S.G. Sotocinal, R.E. Sorge, A. Zaloum, A.H. Tuttle, L.J. Martin, J.S. Wieskopf, J.C. Mapplebeck, P. Wei, S. Zhan, S. Zhang, J.J. McDougall, O.D. King, J.S. Mogil, The Rat Grimace Scale: a partially automated method for quantifying pain in the laboratory rat via facial expressions, Mol. Pain 7 (2011) 55.
	Y. Nagakura, T. Oe, T. Aoki, N. Matsuoka, Biogenic amine depletion causes chronic muscular pain and tactile allodynia accompanied by depression: A putative animal model of fibromyalgia, Pain 146 (2009), 26–33.
Resource availability:	

## Method details

### Animals

All animal experiments and care were conducted in accordance with institutional guidelines that are in compliance with the Fundamental Guidelines for Proper Conduct of Animal Experiments and Related Activities in Academic Research Institutions under the jurisdiction of the Ministry of Education, Culture, Sports, Science, and Technology (Notice No. 71, Japan, 2006), and all experimental procedures were approved by the Institutional Animal Care and Use Committee of Aomori University (Aomori, Japan). Male Sprague–Dawley rats weighing 200–250 g were purchased from Clea Japan, Inc. (Tokyo, Japan). Animals were individually housed in acrylic cages with woodchips as bedding, a 12 h light/dark cycle with controlled temperature (23±2 °C) and humidity (55±10%), and access to standard laboratory chow and water ad libitum. All tests were conducted during the light cycle (08:00–20:00 h). Animals were allocated to experimental groups in such a way that the groups were comparable in terms of body weight.

### Induction of the fibromyalgia-like condition in rats

A previously optimized regimen of reserpine, i.e., 1 mg/kg, subcutaneous, once daily for 3 consecutive days [1], was used to induce a fibromyalgia-like condition in rats. Reserpine (Nacalai Tesque, Kyoto, Japan) was weighed, transferred to a 1.5 mL microtube, and dissolved in glacial acetic acid (FUJIFILM Wako Pure Chemical Co., Osaka, Japan) with sonication. This solution was diluted in a test tube with distilled water, resulting in final concentration of 1 mg/mL reserpine in 0.5% acetic acid. The injection of reserpine was initiated when the body weight of animal was <300 g. The reserpine solution was injected subcutaneously on the back of rats in a volume of 1 mL/kg (i.e., 1 mg reserpine/kg). The injection was administered once daily for 3 consecutive days. Control animals were injected with vehicle (0.5% acetic acid, 1 mL/kg of body weight).

### Videotaping

**Fig. 1:**
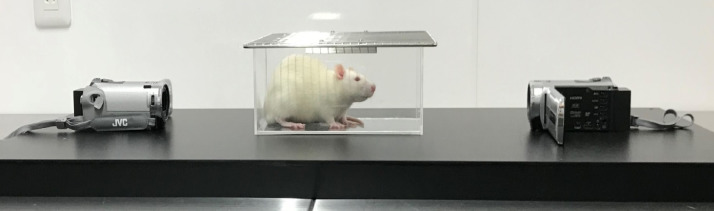
Videotaping of rat facial expression using two high-resolution video cameras.

Each animal was placed in an acrylic chamber (width: 210 mm, depth: 105 mm, height: 90 mm) with a wire-mesh ceiling to allow ventilation and was digitally videotaped for 30 min using two high-resolution (1920 × 1080) color video cameras (Jvc Gz-E242-S, JvcKenwood, Yokohama, Japan) positioned in front and behind the chamber as shown in Fig. 1. In time course experiment, videotaping was conducted in vehicle- or reserpine-injected animals prior to the injection as baseline and on days 3, 5, 7, and 17 after the first injection.

### Image capturing

Still images (Portable Network Graphic format) of each animal were captured at approximately 3 min intervals from each 30 min video file using Windows Media Player and Print Screen functions (Microsoft Co., Redmond, WA) at points where the head of the animal was motionless. Further, the face from the still image was cropped and enlarged. A total of 10 face images of each animal were obtained, including an in-focus view of parts of the face used for scoring (i.e., eyes, nose/cheeks, ears, and whiskers). The observer who captured the images was blinded to the allocation of treatment to rats.

### Image scoring

The captured face images (10 images/rat) were pasted in PowerPoint 2013 (Microsoft Co., Redmond, WA). The images (one image per slide) were presented in random order to three trained raters, who were blinded to the treatment allocation. The raters rated four action units for each image: orbital tightening, nose/cheek flattening, and changes in characteristics of ears and whiskers. For each image, each action unit was rated using a 3-point Likert scale (0 = not present, 1 = moderately present, 2 = obviously present) according to the rating method described by Sotocinal et al. [2]. The average ratings for all four action units were determined. Further, the average of the three raters was calculated; this value was used as the rat grimace scale (RGS) score for each image. Finally, an RGS score for each animal was calculated using the average of the RGS scores of all 10 images for each rat.

### Images for reference

**Fig. 2:**
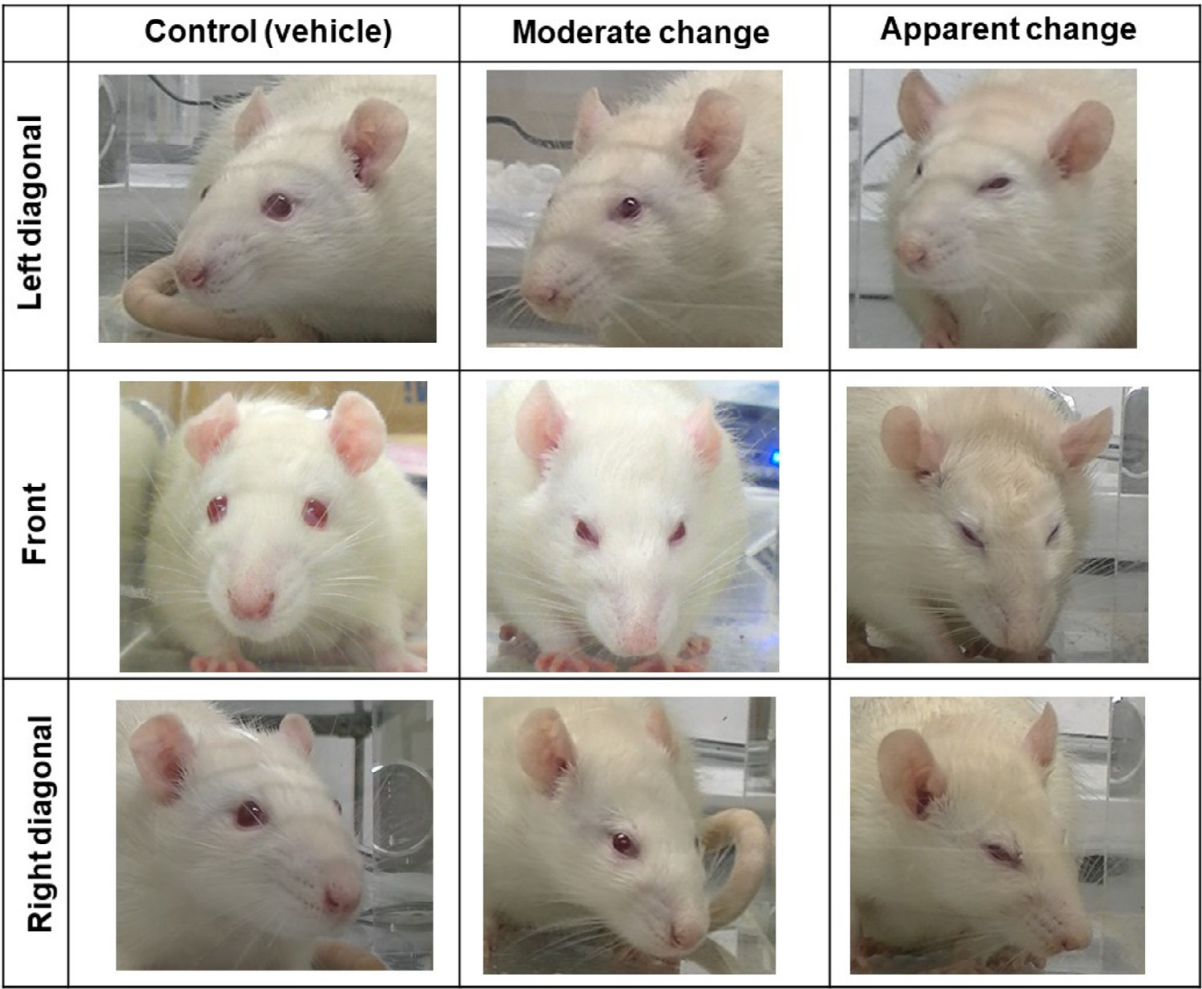
Representative images of facial expression in the initial experiments. Facial expressions in the control (vehicle-treated) rats are represented in the left column. Moderate and apparent facial expression changes appearing in the reserpine-induced myalgia rat were represented in the middle and right columns, respectively.

Preliminary experiments were conducted to collect a representative image set of facial expressions for both the control (vehicle-treated) rats and the reserpine-induced fibromyalgia-like rats. This image set was used as a reference for raters. The representative images are shown in Fig. 2.

### Training material for scoring

Images representing moderate facial expression changes in reserpine-induced fibromyalgia-like rats and control images in vehicle-treated rats were obtained from the videotapes taken on days 3, 5, and 7 after the first injection of reserpine or vehicle, and used as sample images for training of raters. A set of images containing 15 images each for control (vehicle-treated) and reserpine-induced fibromyalgia-like rats is provided as a PowerPoint file (File 1). Images (one per slide) were presented in a random order to three treatment-allocation-blinded raters. Table 1 shows the scores of four separate action units for each image rated by three raters. Interrater reliability among the three raters was determined using two-way single-score intraclass correlation coefficients (ICC) analysis. Table 2 shows the ICC and 95% confidence interval (CI) for action unit scores rated by the three raters. Table 3 summarizes the average values for control and reserpine-induced fibromyalgia-like rats from each rater. Statistically significant differences between the control rats and reserpine-induced fibromyalgia-like rats were assessed for all three raters by the Mann-Whitney *U* test.

**Table 1:** Action unit scores for the images presented in File 1.

		Rater 1					Rater 2					Rater 3				
Slide No.	Treatment	Eye	Nose/cheek	Ear	Whisker	Average (4 units)	Eye	Nose/cheek	Ear	Whisker	Average (4 units)	Eye	Nose/cheek	Ear	Whisker	Average (4 units)
1	Reserpine	2	2	2	2	2	2	2	2	1	1.75	1	1	1	1	1
2	Control	0	0	0	1	0.25	0	0	0	1	0.25	0	0	0	0	0
3	Reserpine	1	1	1	1	1	1	1	1	2	1.25	1	1	1	1	1
4	Reserpine	1	1	1	1	1	1	1	0	2	1	1	1	0	1	0.75
5	Control	0	0	0	0	0	0	0	0	0	0	1	0	0	0	0.25
6	Control	1	0	1	0	0.5	0	0	0	0	0	1	0	0	0	0.25
7	Reserpine	2	2	2	1	1.75	2	2	2	2	2	2	2	2	1	1.75
8	Control	0	1	0	0	0.25	0	2	1	1	1	0	1	0	0	0.25
9	Reserpine	1	2	1	1	1.25	1	2	1	2	1.5	1	1	1	1	1
10	Control	1	0	1	0	0.5	1	1	1	1	1	1	1	1	1	1
11	Reserpine	2	2	2	1	1.75	2	2	2	2	2	2	2	2	2	2
12	Control	0	0	0	0	0	0	0	0	0	0	0	0	0	0	0
13	Reserpine	1	0	1	1	0.75	1	1	1	2	1.25	1	0	1	1	0.75
14	Control	0	0	0	0	0	0	0	0	0	0	0	0	0	0	0
15	Control	0	0	1	0	0.25	0	0	1	0	0.25	0	0	0	1	0.25
16	Reserpine	2	1	1	1	1.25	2	2	2	2	2	2	2	1	2	1.75
17	Control	1	1	1	0	0.75	1	0	1	0	0.5	1	1	1	0	0.75
18	Reserpine	1	1	1	0	0.75	1	1	0	0	0.5	1	1	1	1	1
19	Control	0	0	0	0	0	1	0	0	0	0.25	1	1	0	0	0.5
20	Reserpine	2	2	2	1	1.75	2	2	2	2	2	2	2	1	1	1.5
21	Control	0	0	0	0	0	0	0	0	0	0	0	0	0	0	0
22	Reserpine	0	0	1	0	0.25	1	1	1	1	1	1	2	1	1	1.25
23	Reserpine	0	0	1	1	0.5	1	1	0	1	0.75	0	1	1	0	0.5
24	Control	0	0	1	1	0.5	1	1	2	2	1.5	1	1	1	1	1
25	Reserpine	1	1	1	1	1	1	1	0	0	0.5	1	1	2	1	1.25
26	Control	1	2	2	2	1.75	1	2	2	1	1.5	1	1	1	1	1
27	Reserpine	1	0	1	0	0.5	1	1	2	0	1	1	0	0	0	0.25
28	Control	1	0	1	0	0.5	2	1	1	2	1.5	1	1	1	0	0.75
29	Control	0	0	0	0	0	0	0	0	0	0	0	0	0	0	0
30	Reserpine	1	0	1	0	0.5	1	1	1	1	1	1	2	1	1	1.25

**Table 2:** Interclass correlation coefficient (ICC) and 95% confidence interval (CI) for action unit scores rated by the three raters.

	Eye		Nose/cheek		Whisker		Ear		Average (4 units)	
	Control	Reserpine	Control	Reserpine	Control	Reserpine	Control	Reserpine	Control	Reserpine
ICC (3,1)	0.646	0.807	0.610	0.506	0.427	0.395	0.752	0.345	0.707	0.667
95% CI	0.610 - 0.923	0.365 - 0.848	0.318 - 0.830	0.193 - 0.773	0.108 - 0.725	0.076 - 0.704	0.520 - 0.899	0.028 - 0.670	0.451 - 0.878	0.394 - 0.859
*F*	6.471	13.512	5.688	4.079	3.239	2.957	10.097	2.578	8.227	7.013
*P*	< 0.001	< 0.001	< 0.001	< 0.001	0.004	0.007	< 0.001	0.016	< 0.001	< 0.001

**Table 3:** Average action unit scores of control rats and reserpine-induced fibromyalgia-like rats for each rater.

		Rater 1					Rater 2					Rater 3				
		Eye	Nose/cheek	Ear	Whisker	Average (4 units)	Eye	Nose/cheek	Ear	Whisker	Average (4 units)	Eye	Nose/cheek	Ear	Whisker	Average (4 units)
Control (*n* = 15)	Averege	0.33	0.27	0.53	0.27	0.35	0.47	0.47	0.60	0.53	0.52	0.53	0.47	0.33	0.27	0.40
	Standard deviation	0.03	0.04	0.04	0.04	0.03	0.04	0.05	0.05	0.05	0.04	0.03	0.03	0.03	0.03	0.03
Reserpine (*n* = 15)	Averege	1.20⁎⁎	1.00*	1.27⁎⁎	0.80⁎⁎	1.07⁎⁎	1.33⁎⁎	1.40⁎⁎	1.13	1.33*	1.30⁎⁎	1.27⁎⁎	1.27⁎⁎	1.07⁎⁎	1.00⁎⁎	1.15⁎⁎
	Standard deviation	0.05	0.06	0.04	0.04	0.04	0.04	0.04	0.06	0.06	0.04	0.04	0.05	0.04	0.04	0.03

⁎ *P*< 0.05,.

⁎⁎ *P*< 0.01, statistically significant difference compared to control group by Mann–Whitney *U* test.

### Time course of the RGS change

Time course experiment was conducted using 6 animals each for vehicle (control) and reserpine treatment. Table 4 shows the average RGS scores for the 6 animals prior to the vehicle or reserpine injection as baseline and on days 3, 5, 7, and 17 after the first injection. The RGS score for reserpine-induced fibromyalgia-like rats was significantly higher compared to that for control rats even on day 17.

**Table 4:** Time course of the RGS scores associated with injection of reserpine.

		Prior to injection	day 3	day 5	day 7	day 17
Control (*n* = 6)	Averege	0.19	0.30	0.24	0.38	0.38
	Standard deviation	0.04	0.12	0.03	0.08	0.13
Reserpine (*n* = 6)	Averege	0.19	1.56	1.11	1.05	0.84⁎⁎
	Standard deviation	0.03	0.17	0.39	0.07	0.24

⁎⁎ *P*< 0.01, statistically significant difference compared to control group.

## Additional information

Nociplastic pain can occur without organic causes (e.g., inflammation or nerve damage) and is associated with various clinical disorders, including fibromyalgia [3]. It is emerging as a serious healthcare issue affecting those who have the disorder as well as social welfare [4]. Accordingly, preclinical research should contribute to the development of better therapies, where animal models are used for assessing the efficacy of therapeutic compounds under development. Because pain occurs in patients without an external stimulus, it is essential to assess spontaneous pain in animals [5]. The grimace scale was developed as a methodology for rating facial expression [6] and has been widely cited as a method for measuring spontaneous pain in animals [7], [8], [9]. However, the grimace scale score change in animals has been short-lived; thus, it is only useful in assessing pain in the early phase [8]. Furthermore, the grimace scale has never been applied to animals exhibiting nociplastic pain. Here, we applied the RGS reported by Sotocinal et al. [2]. to the reserpine-induced fibromyalgia-like rat, which manifests nociplastic pain caused by chronic depletion of endogenous monoamines [1] and demonstrated that a substantial and long-lasting elevation of RGS scores occurred in such rats. The measurement of RGS score in the reserpine-induced fibromyalgia-like rat simulates spontaneous pain in patients with nociplastic pain and may be related to the translation of efficacies of therapeutic compounds between animals and humans.

## Declaration of Competing Interest

The authors confirm that there are no conflicts of interest.

## Footnotes

Supplementary material associated with this article can be found, in the online version, at doi:10.1016/j.mex.2020.100826.

## References

[1] Nagakura Y., Oe T., Aoki T., Matsuoka N. (2009). Biogenic amine depletion causes chronic muscular pain and tactile allodynia accompanied by depression: a putative animal model of fibromyalgia. Pain.

[2] Sotocinal S.G., Sorge R.E., Zaloum A., Tuttle A.H., Martin L.J., Wieskopf J.S., Mapplebeck J.C., Wei P., Zhan S., Zhang S., McDougall J.J., King O.D., Mogil J.S. (2011). The rat grimace scale: a partially automated method for quantifying pain in the laboratory rat via facial expressions. Mol. Pain.

[3] Woolf C.J. (2010). What is this thing called pain?. J. Clin. Invest..

[4] Nagakura Y. (2015). Challenges in drug discovery for overcoming 'dysfunctional pain': an emerging category of chronic pain. Expert Opin. Drug Discov..

[5] Mogil J.S. (2009). Animal models of pain: progress and challenges. Nat. Rev. Neurosci..

[6] Langford D.J., Bailey A.L., Chanda M.L., Clarke S.E., Drummond T.E., Echols S., Glick S., Ingrao J., Klassen-Ross T., Lacroix-Fralish M.L., Matsumiya L., Sorge R.E., Sotocinal S.G., Tabaka J.M., Wong D., van den Maagdenberg A.M., Ferrari M.D., Craig K.D., Mogil J.S. (2010). Coding of facial expressions of pain in the laboratory mouse. Nat. Methods.

[7] Deuis J.R., Dvorakova L.S., Vetter I. (2017). Methods used to evaluate pain behaviors in rodents. Front. Mol. Neurosci..

[8] Tappe-Theodor T.King, Morgan M.M. (2019). Pros and cons of clinically relevant methods to assess pain in rodents. Neurosci. Biobehav. Rev..

[9] Serizawa K., Tomizawa-Shinohara H., Yasuno H., Yogo K., Matsumoto Y. (2019). Anti-IL-6 receptor antibody inhibits spontaneous pain at the pre-onset of experimental autoimmune encephalomyelitis in mice. Front. Neurol..

