# Supplementary material for: The method simulating spontaneous pain in patients with nociplastic pain using rats with fibromyalgia-like condition
Source: MethodsX. 2020 Feb 26;7:100826. doi: 10.1016/j.mex.2020.100826 (PMC7078388; doi:10.1016/j.mex.2020.100826)
Supplement: Supplementary file 1 [file mmc1.zip › File_1.pptx]

## Slide 1
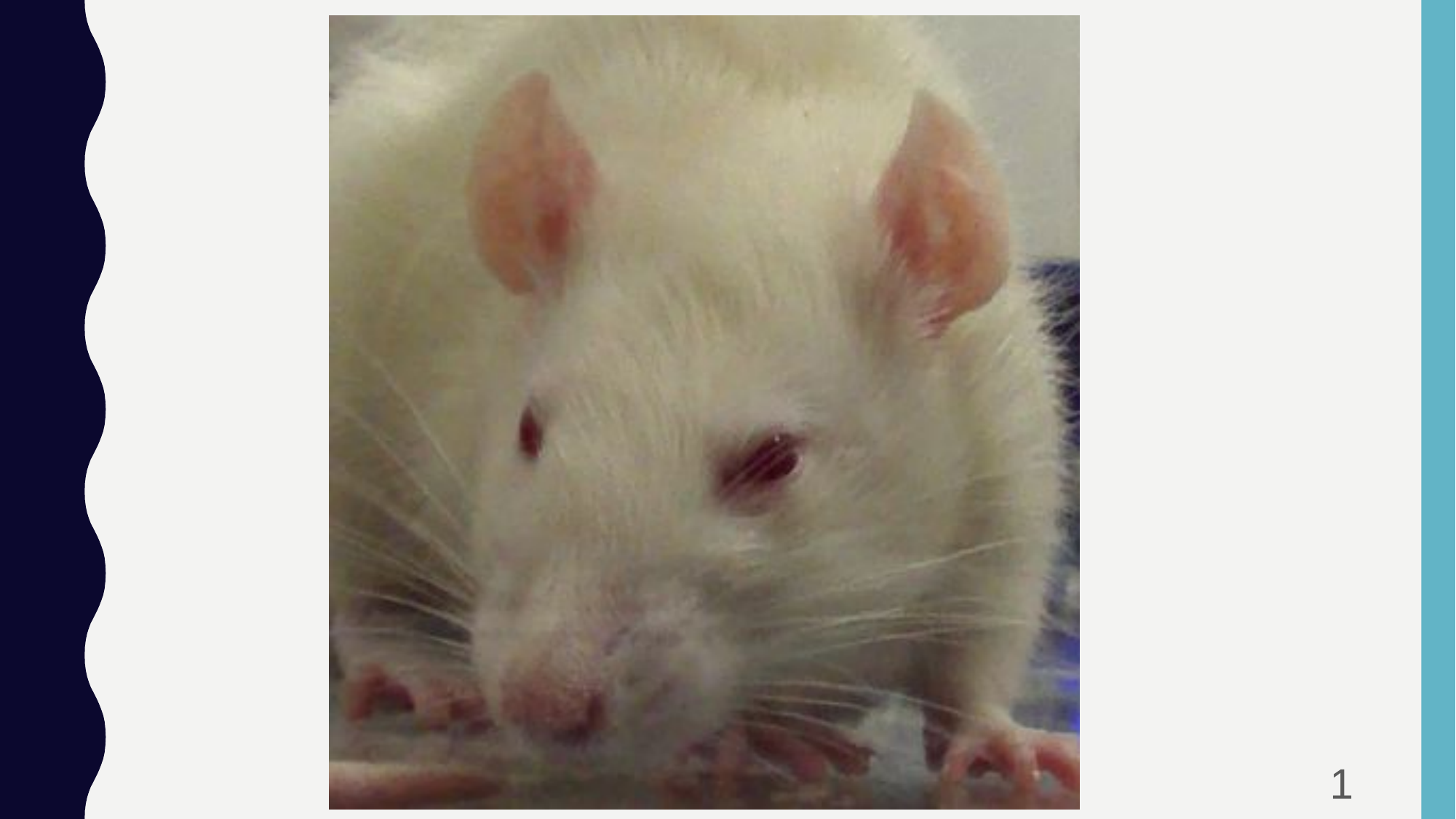

1

## Slide 2
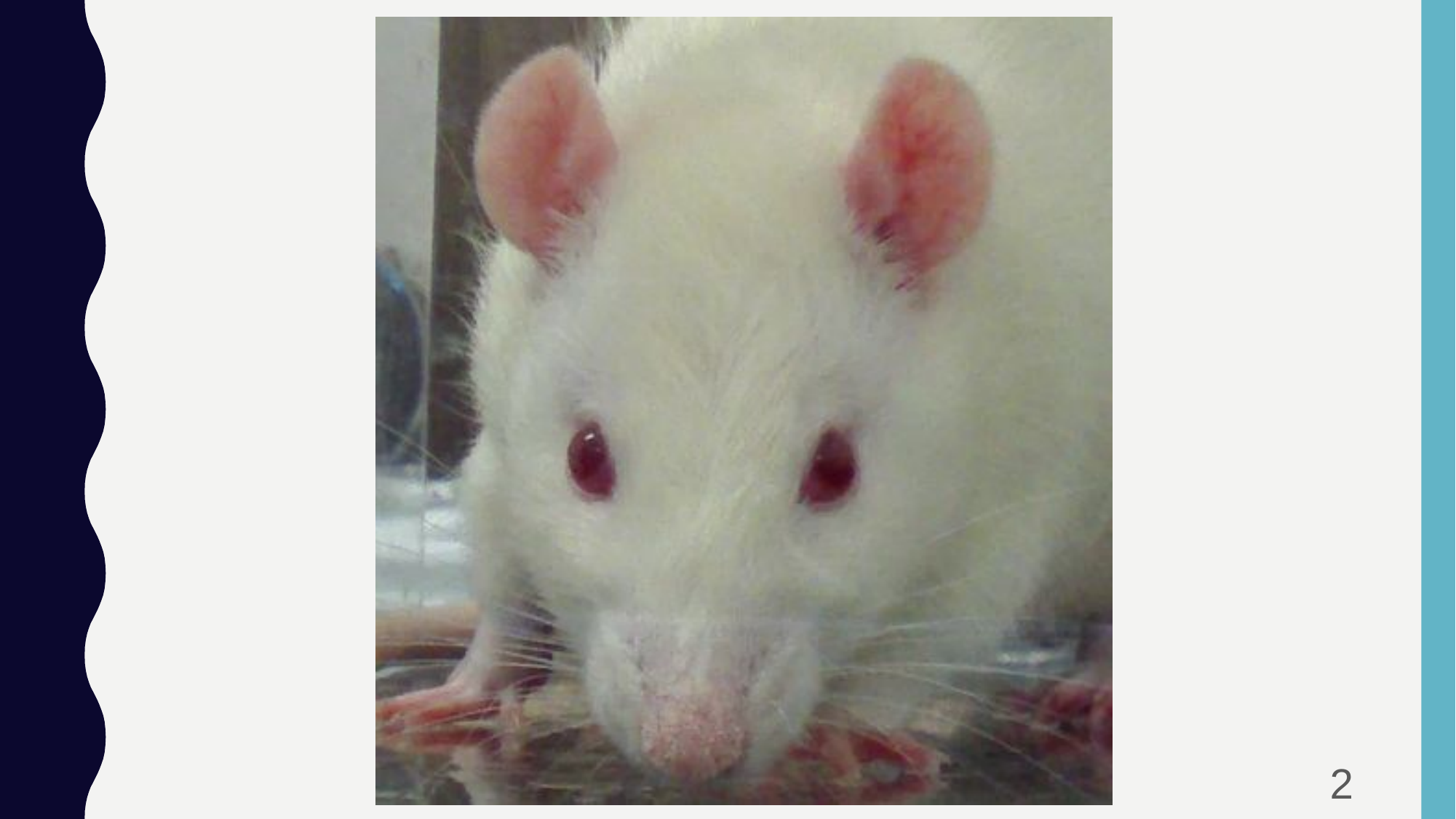

2

## Slide 3
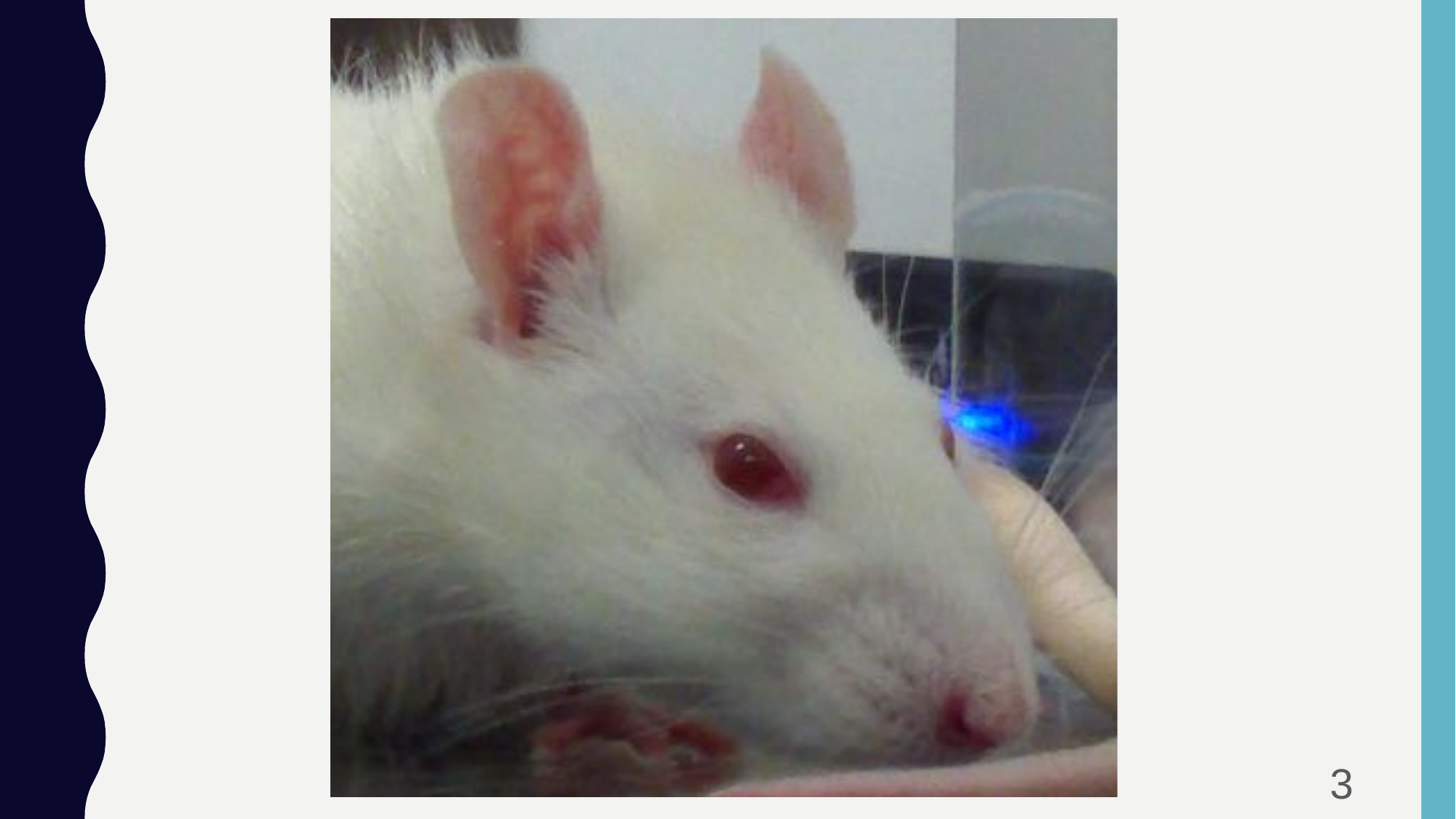

3

## Slide 4
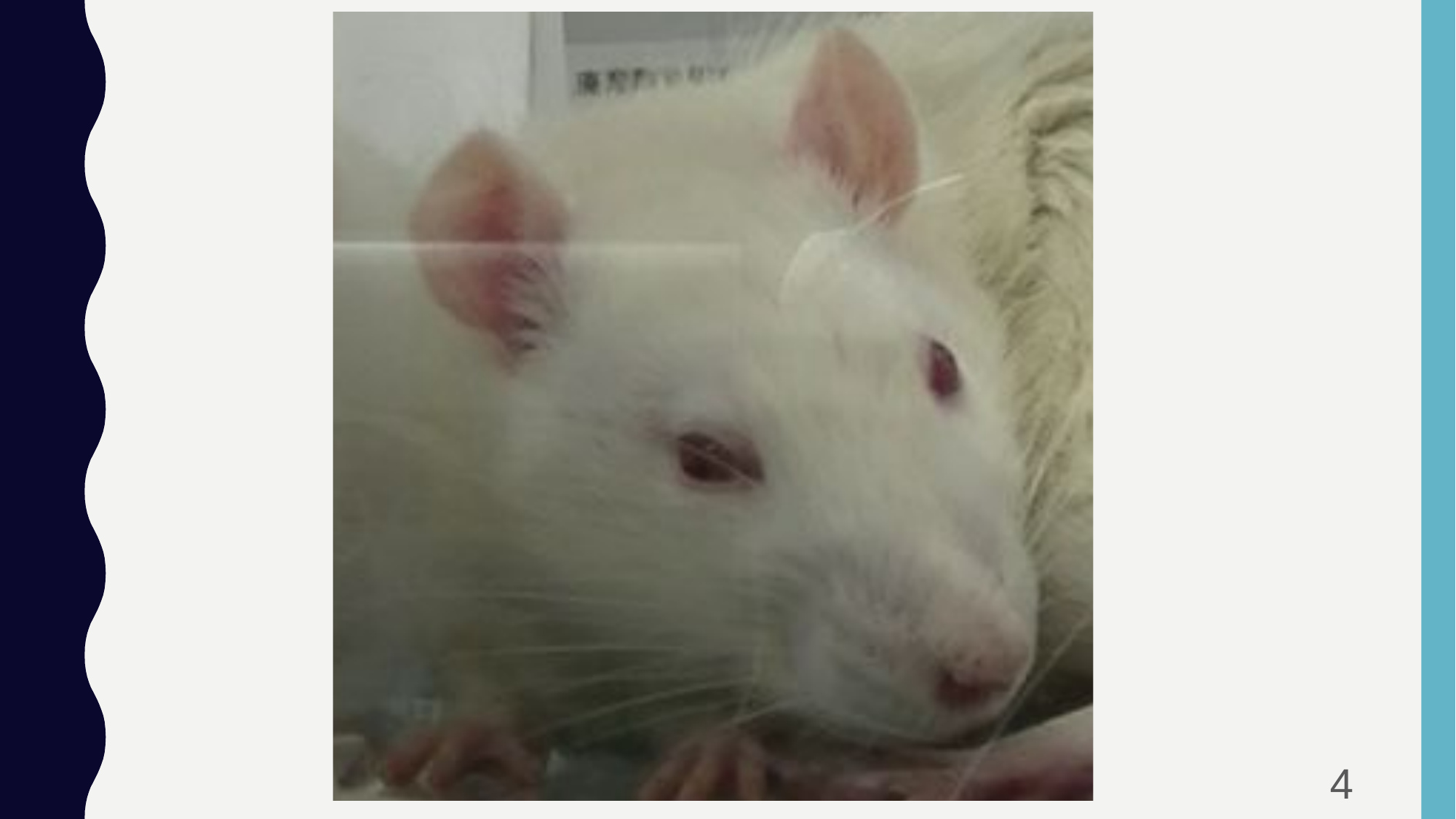

4

## Slide 5
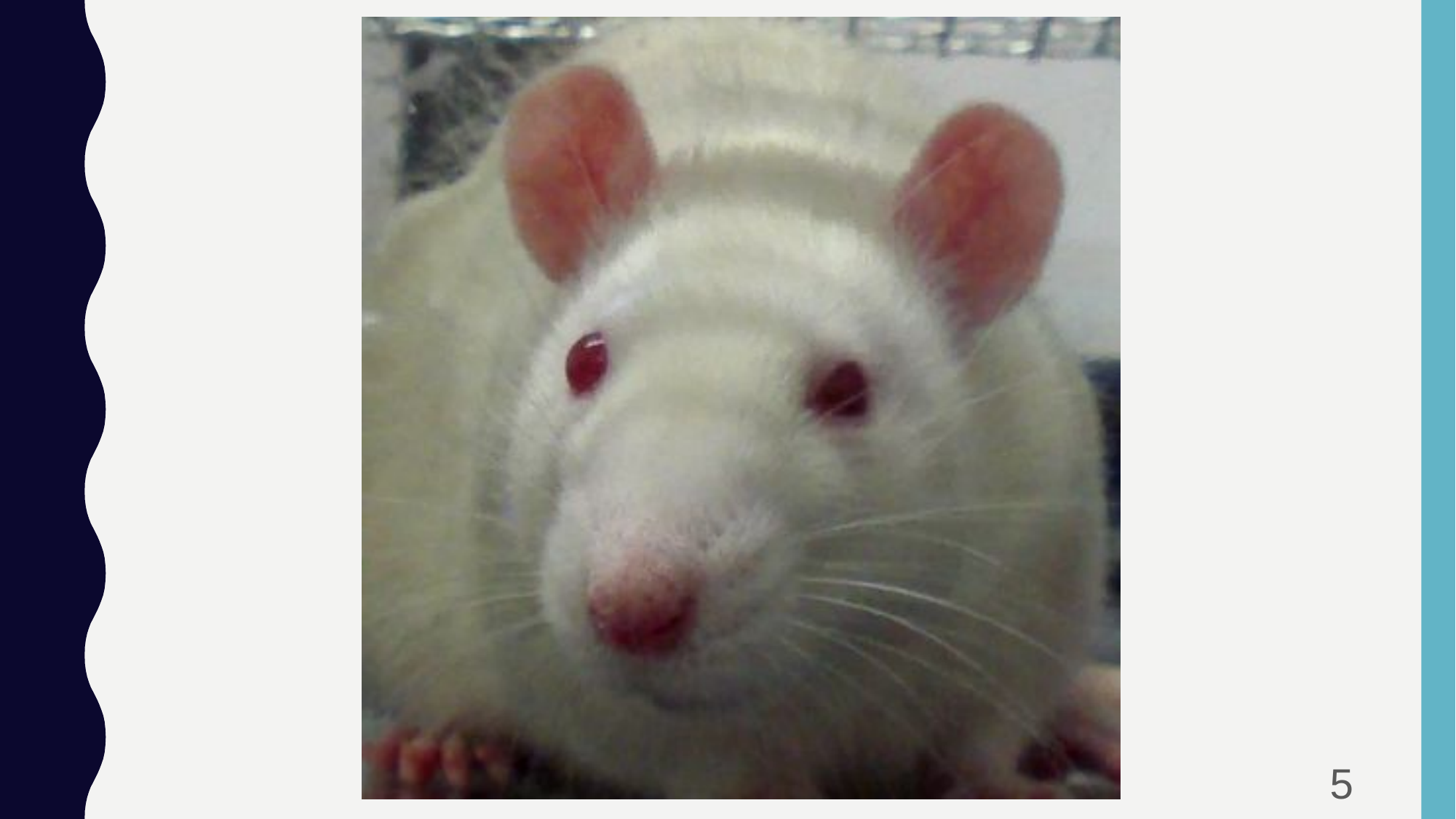

5

## Slide 6
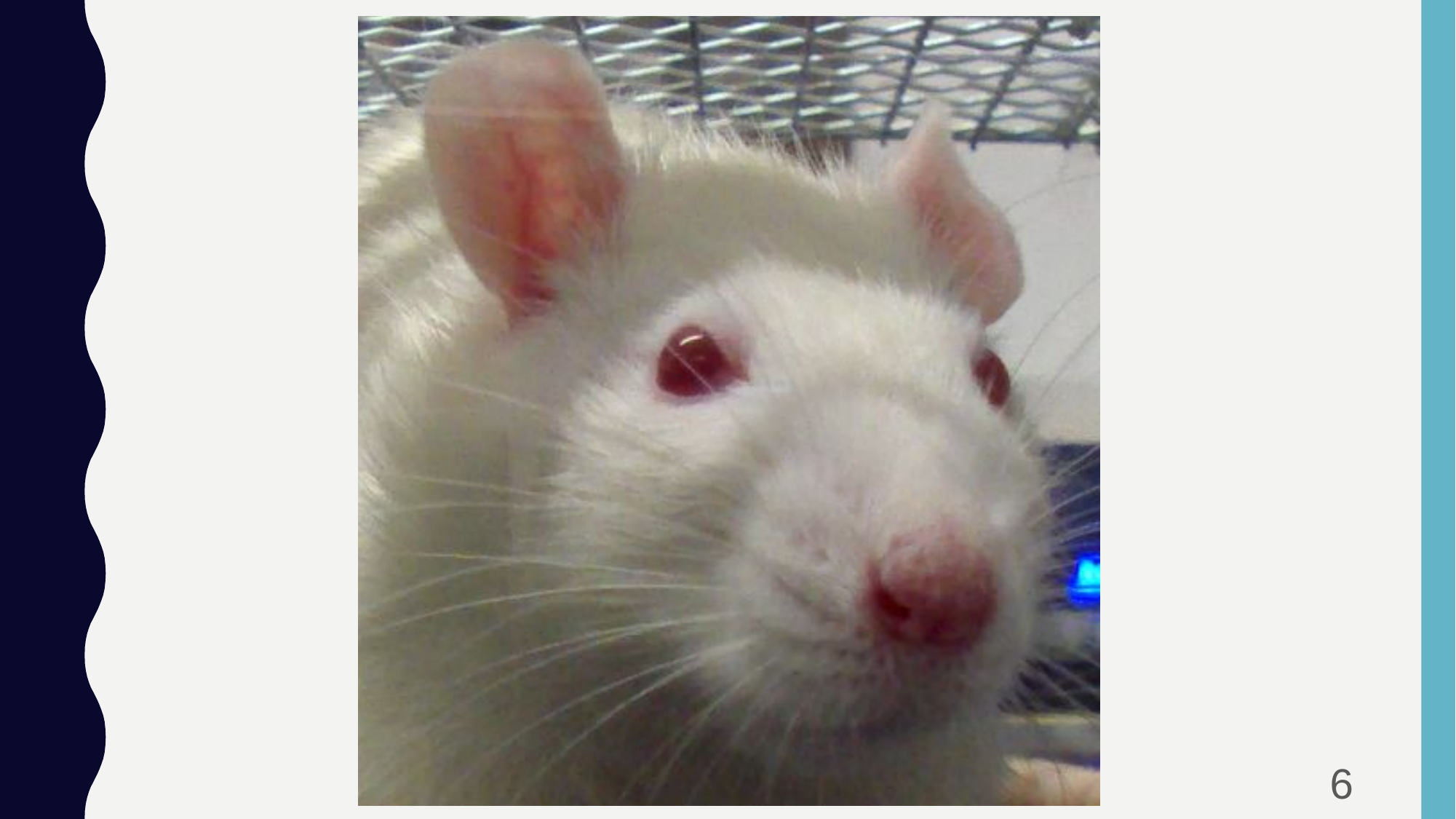

6

## Slide 7
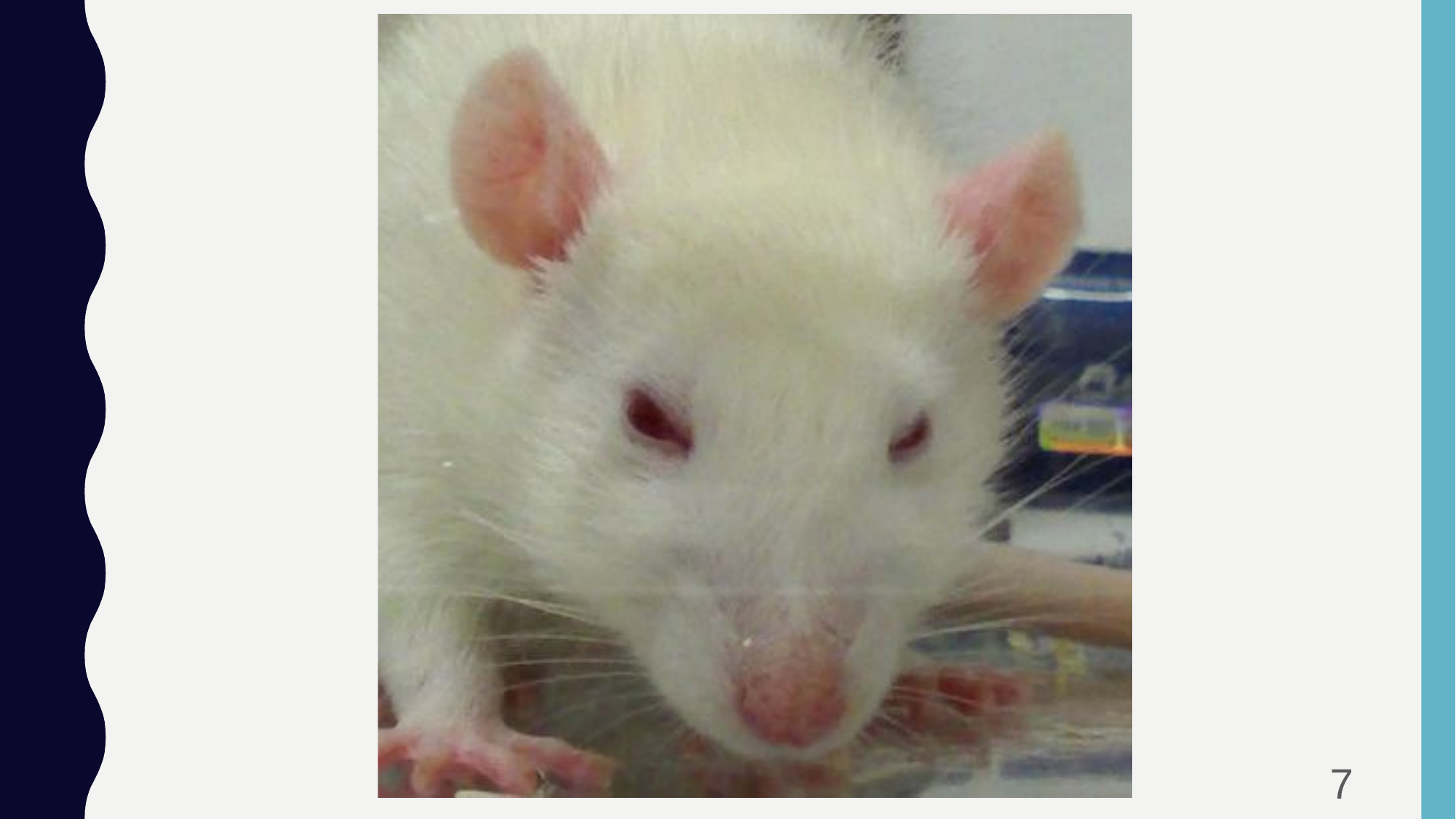

7

## Slide 8
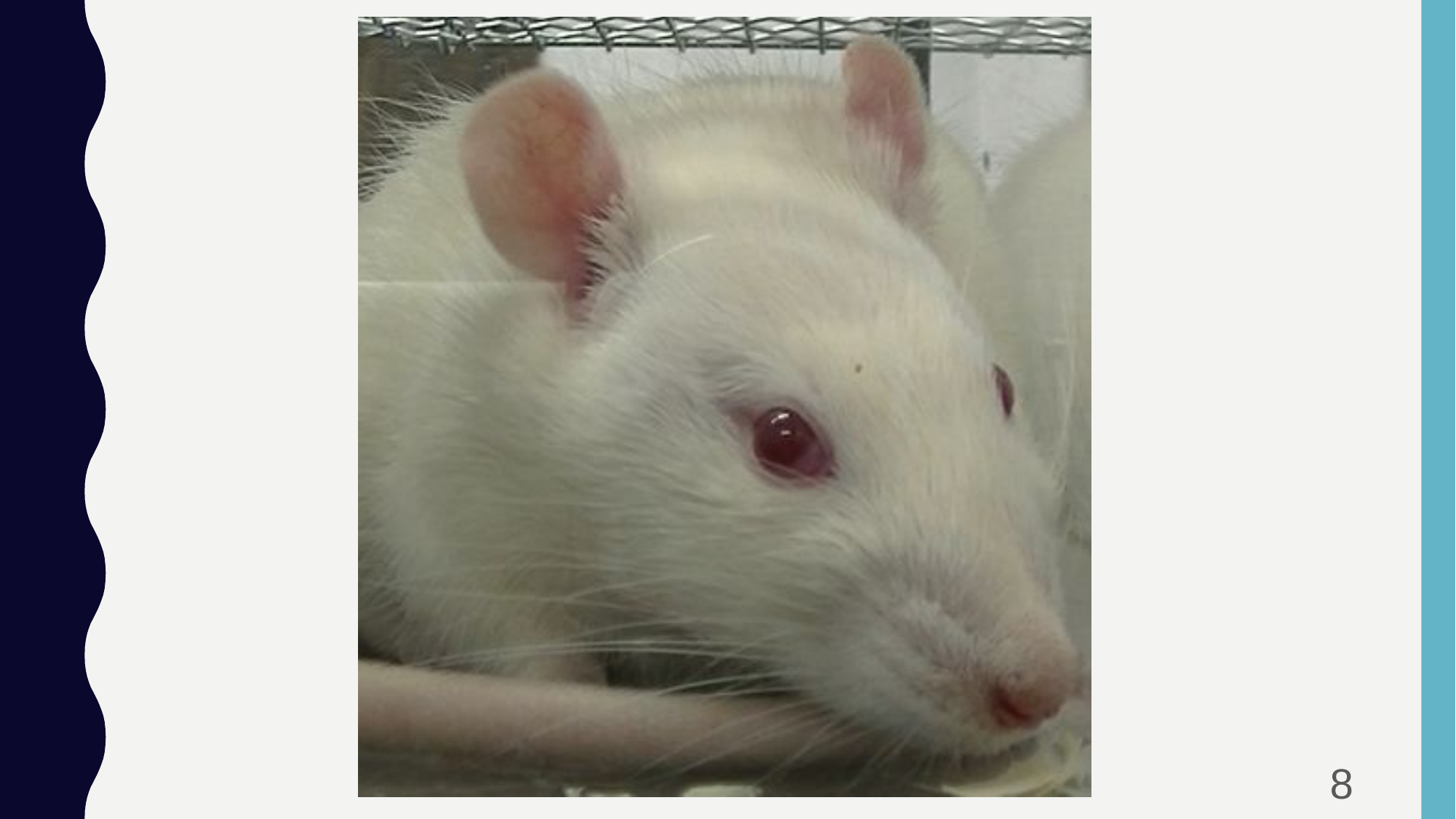

8

## Slide 9
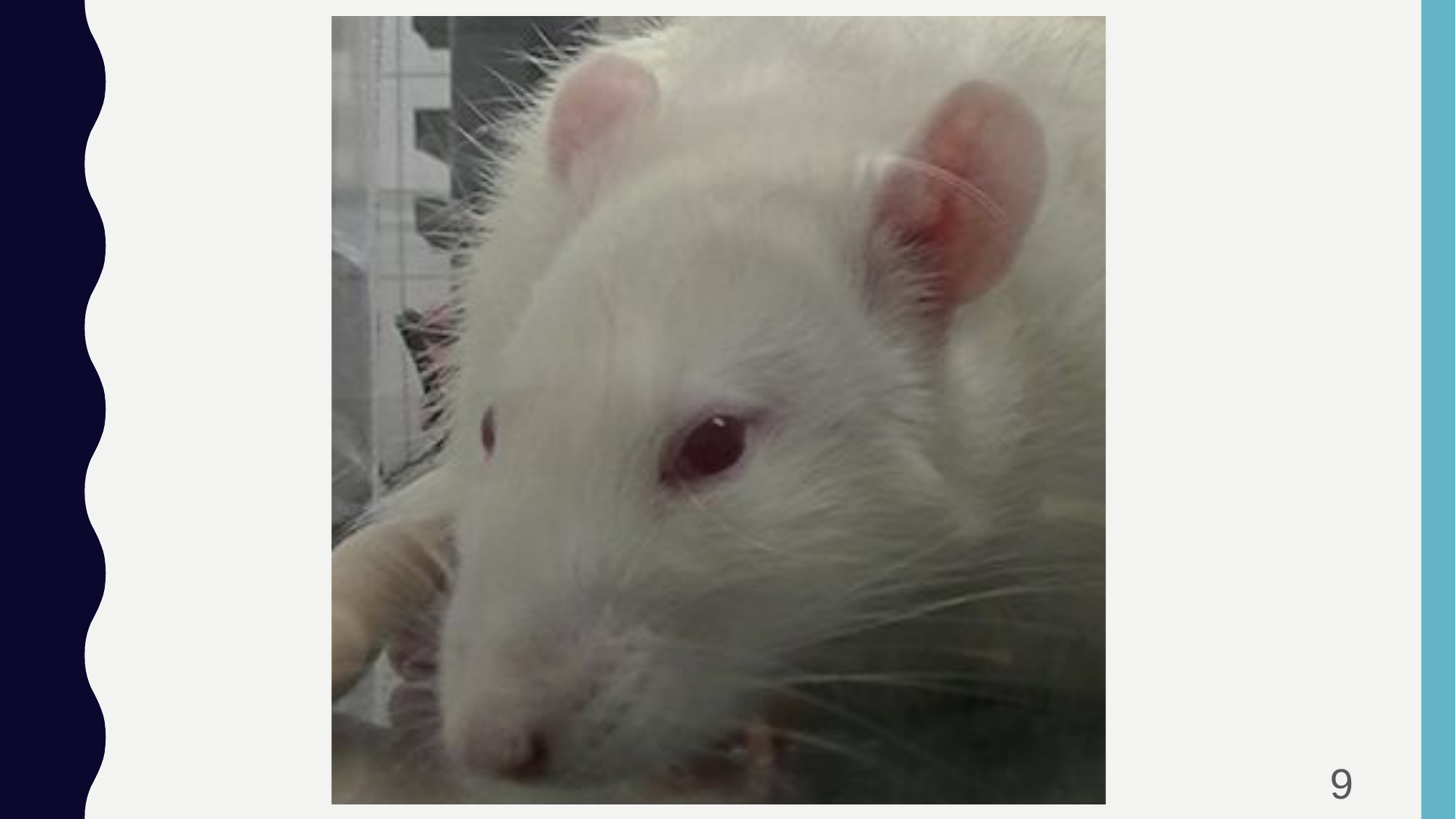

9

## Slide 10
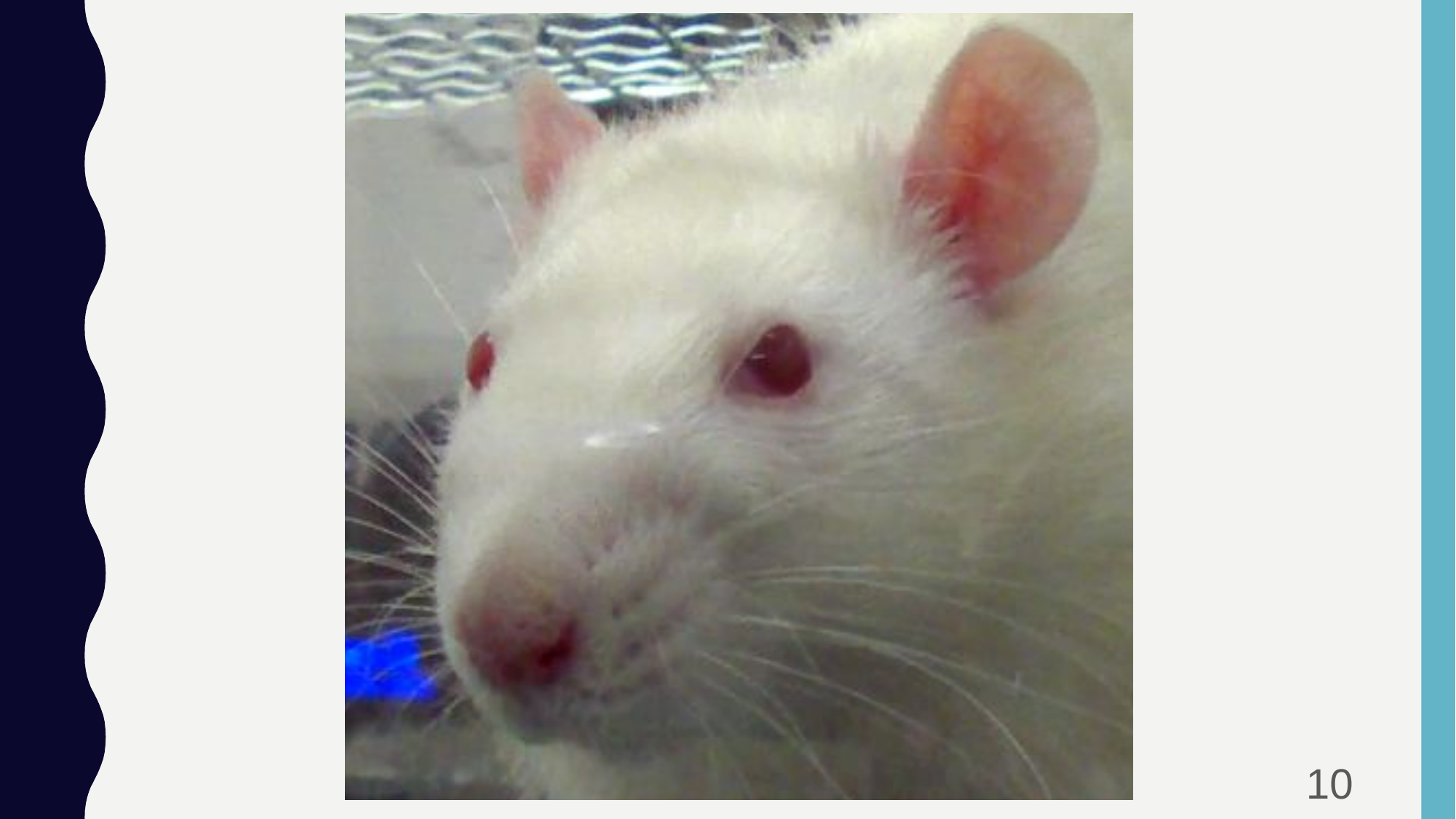

10

## Slide 11
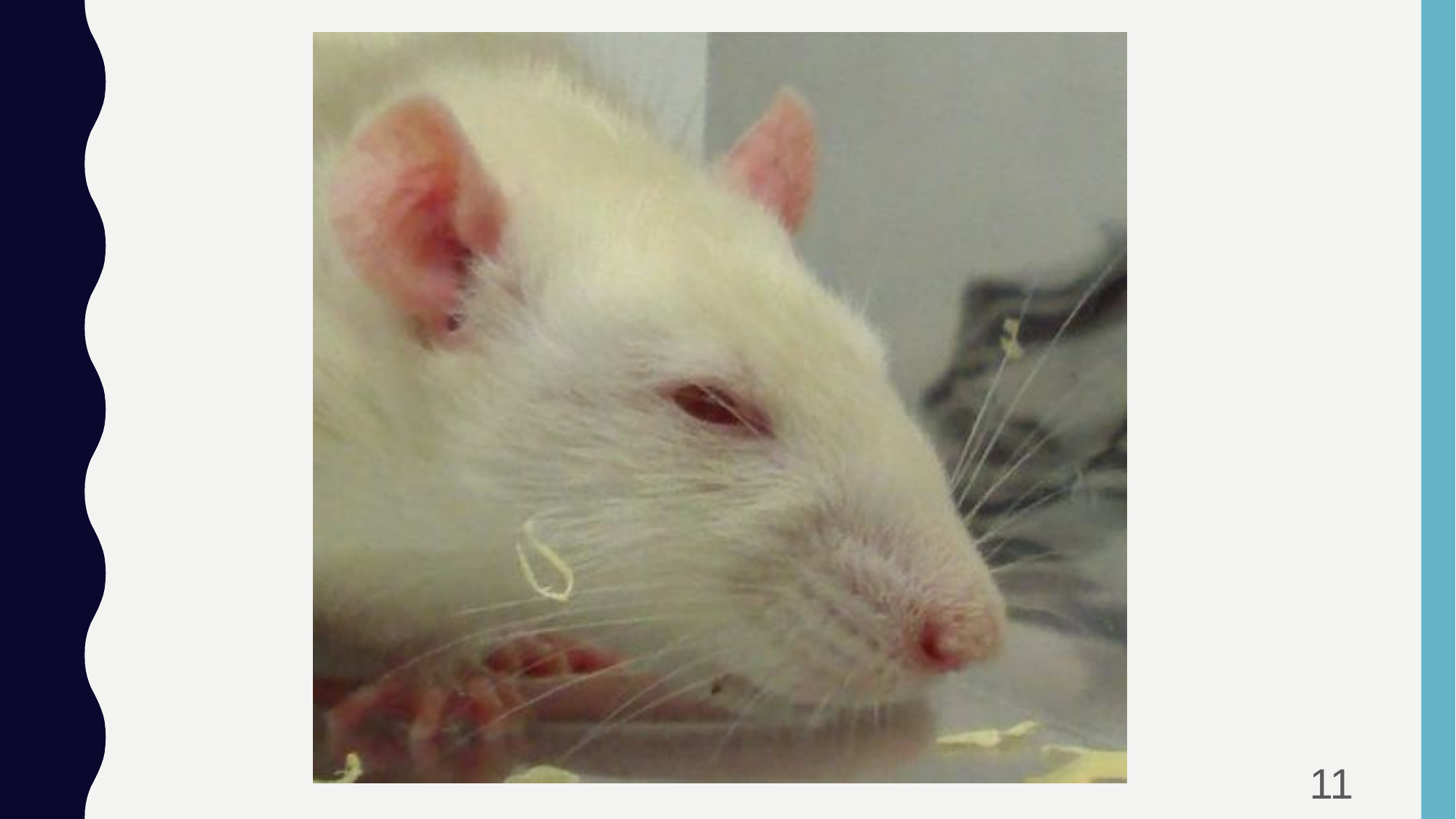

11

## Slide 12
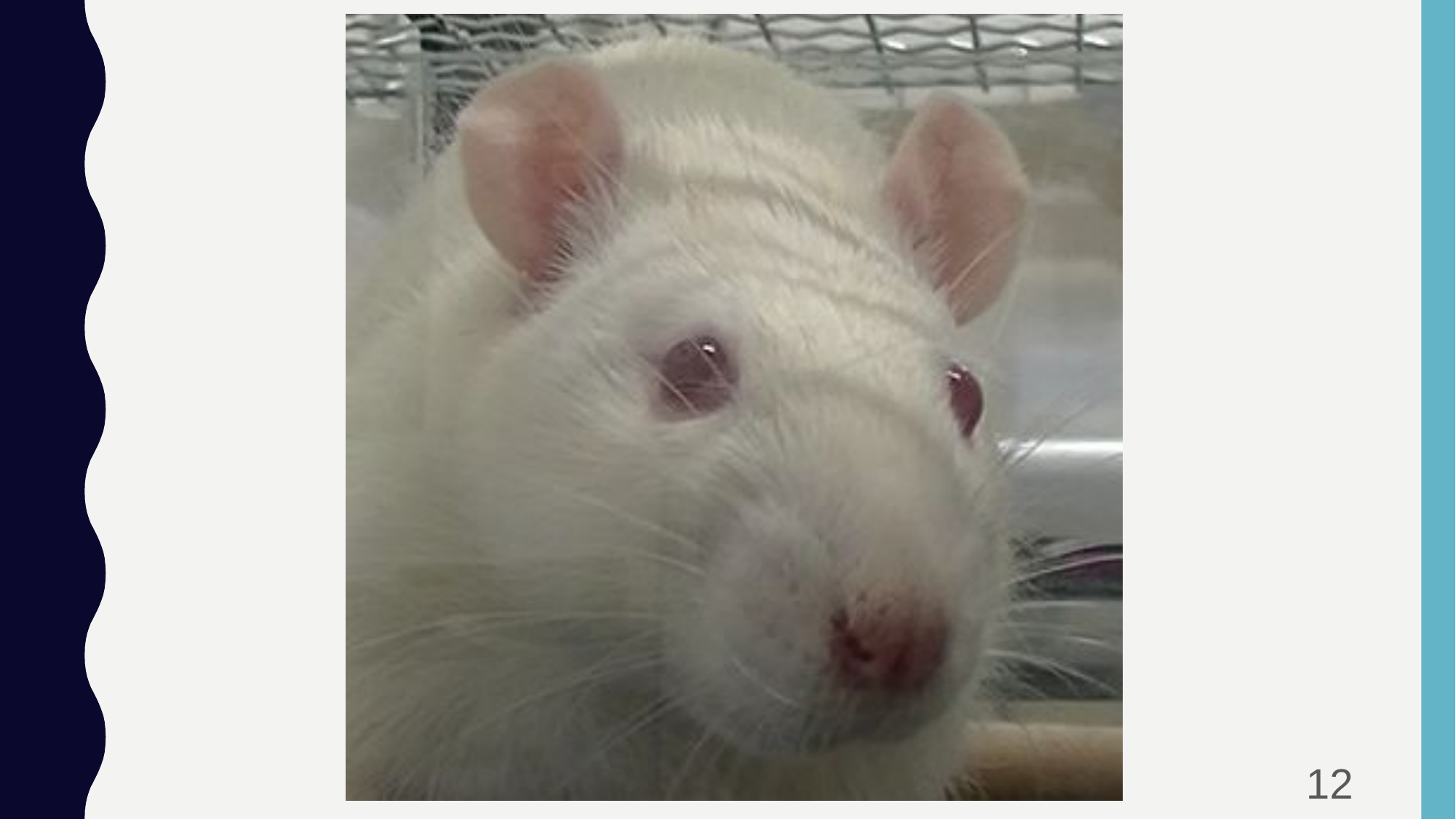

12

## Slide 13
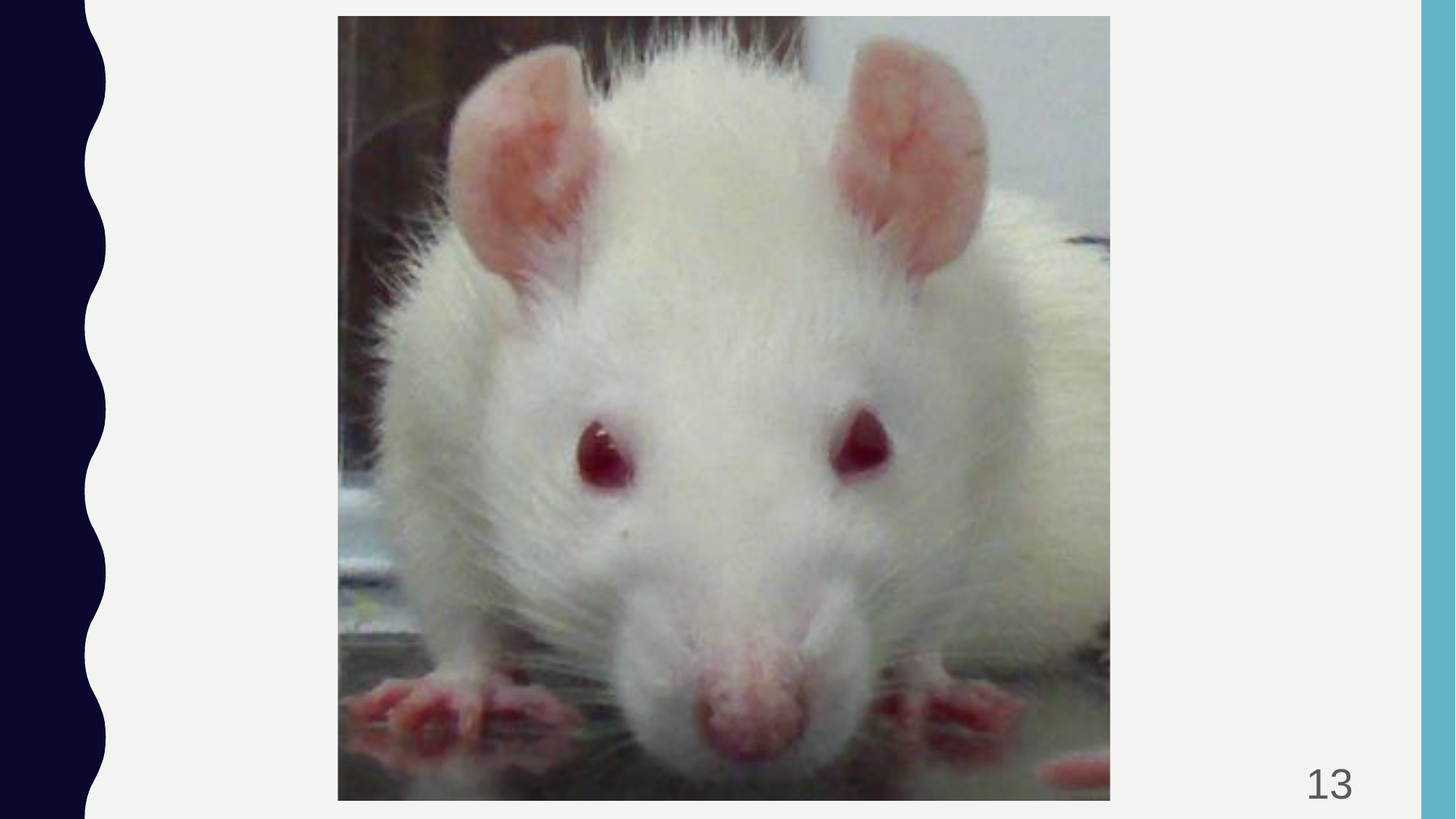

13

## Slide 14
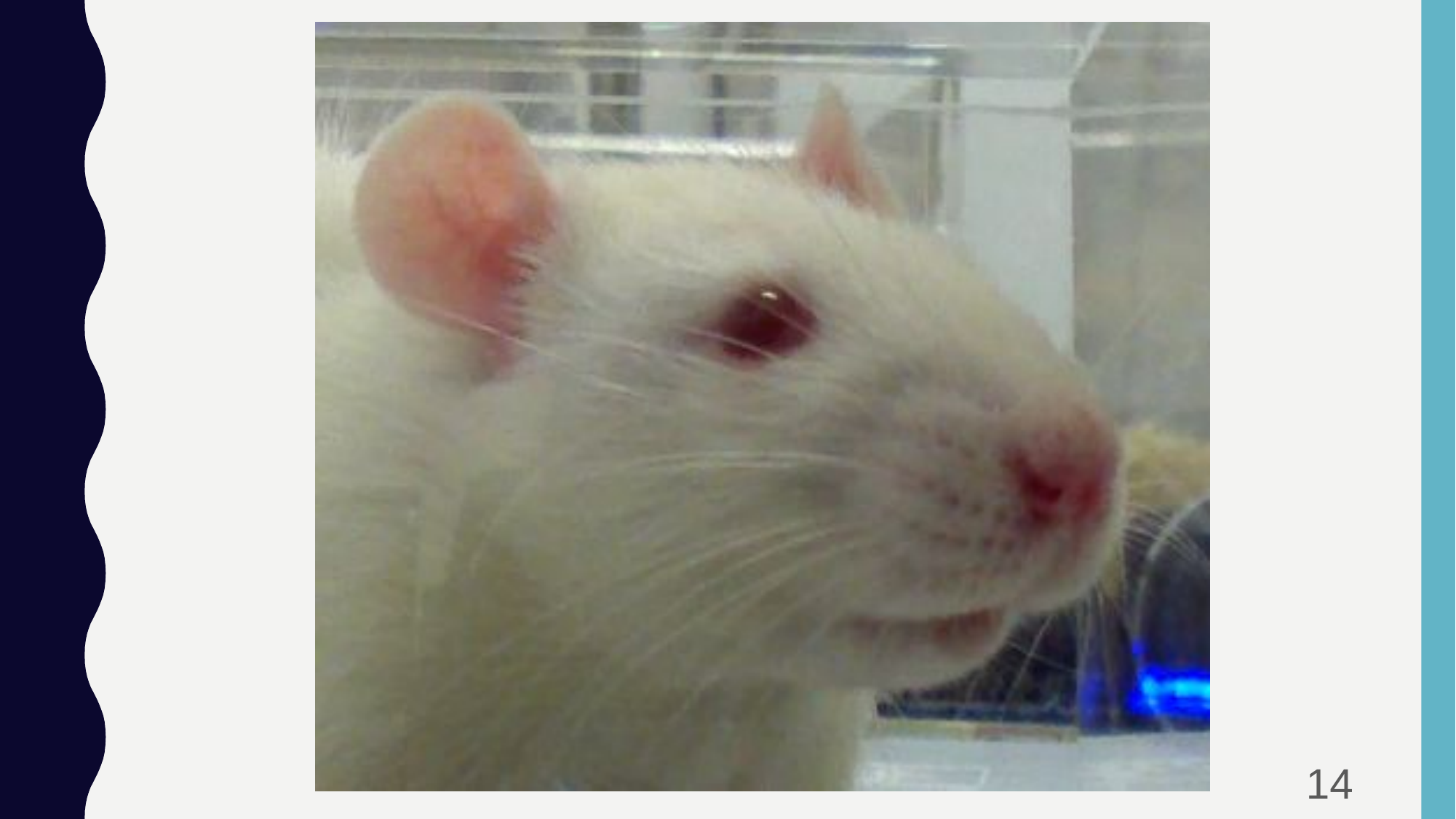

14

## Slide 15
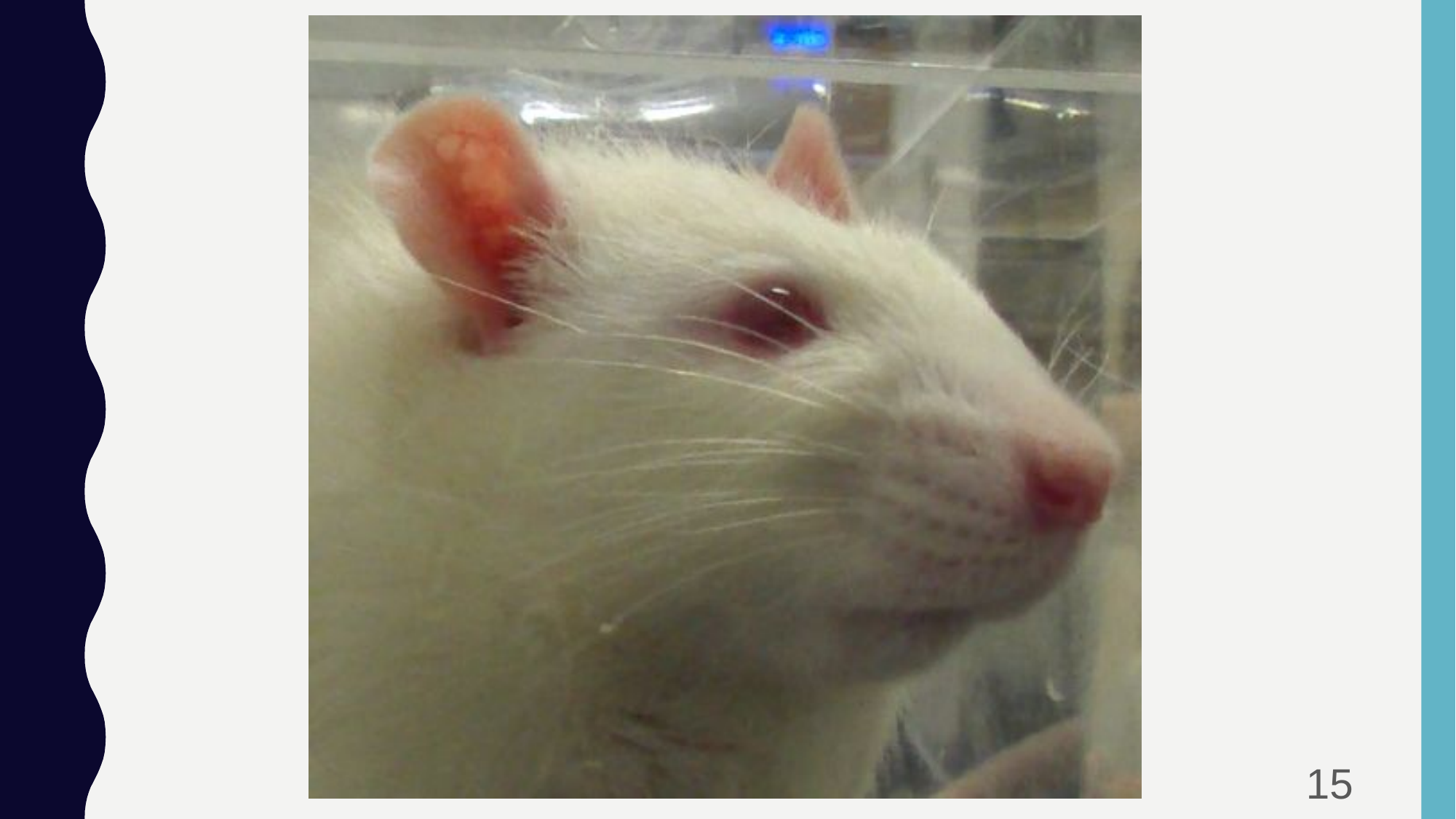

15

## Slide 16
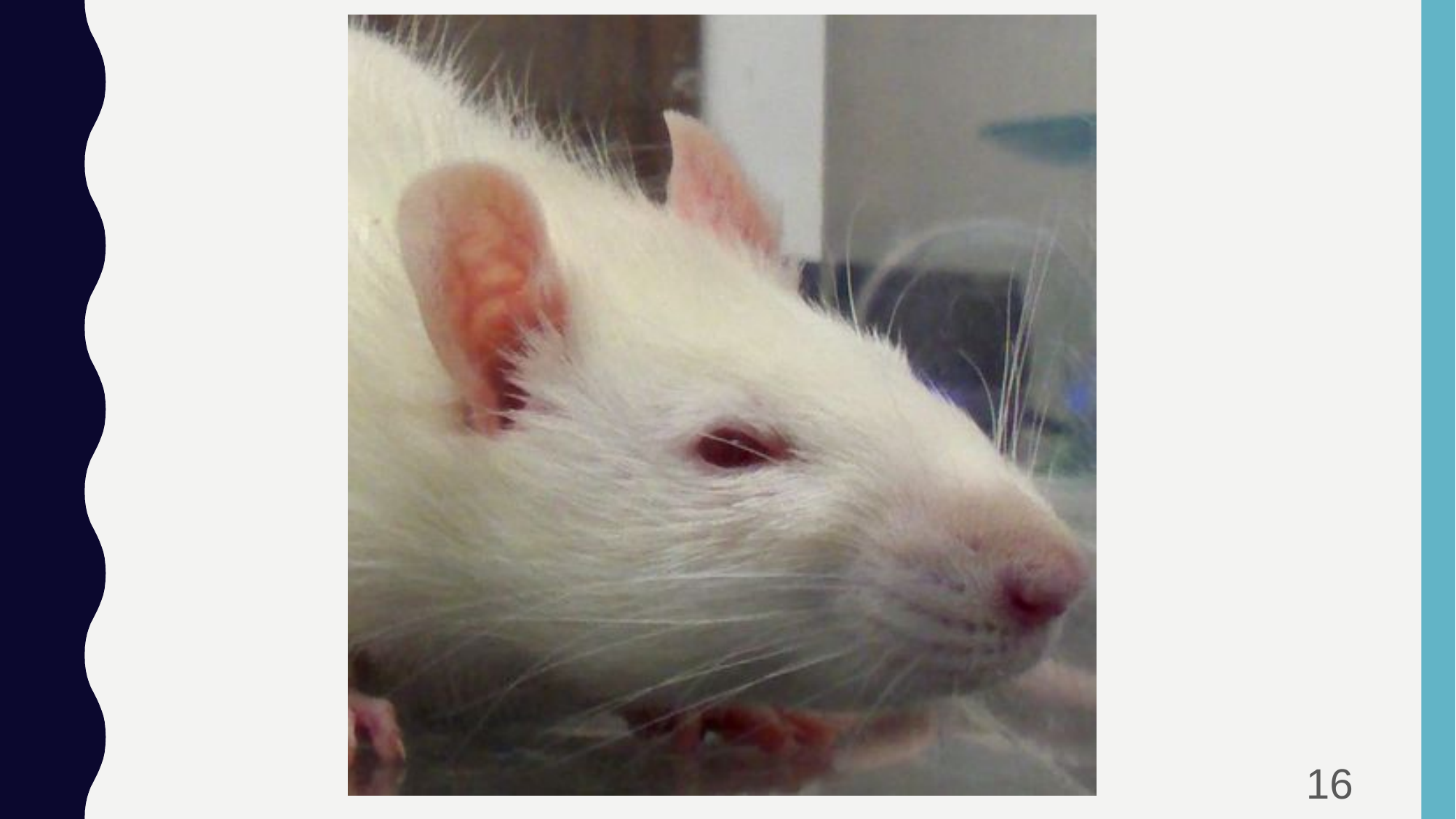

16

## Slide 17
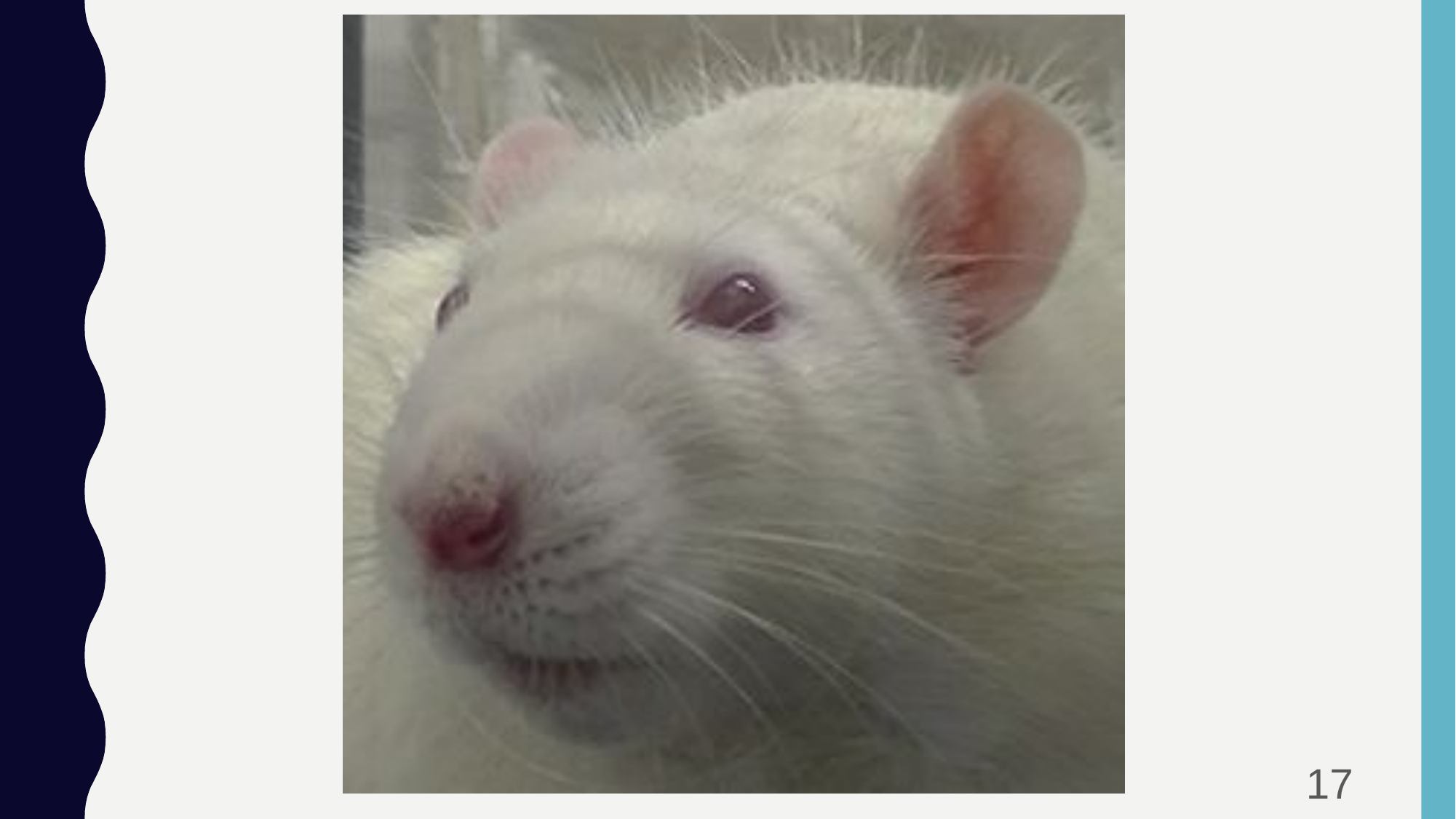

17

## Slide 18
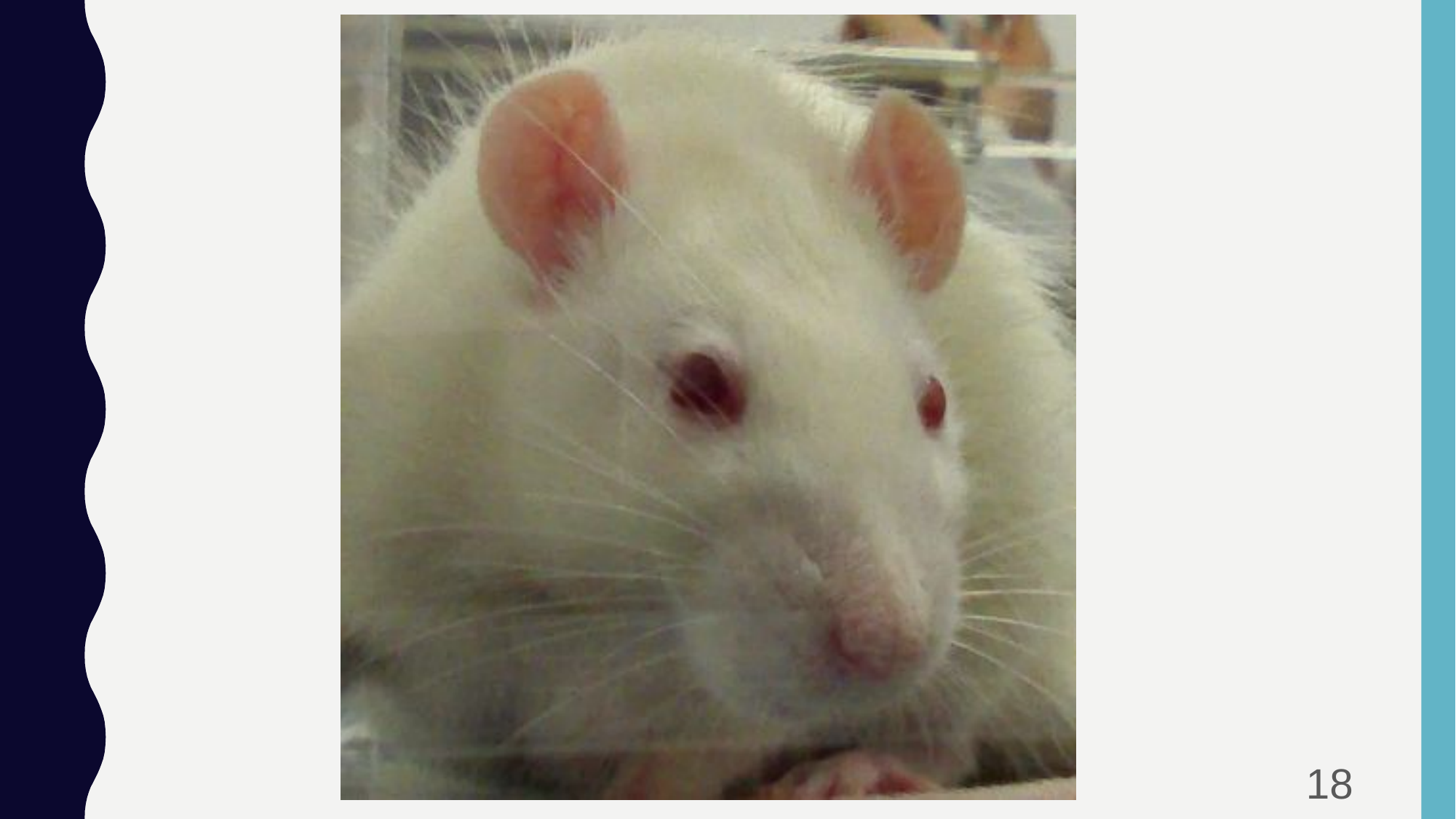

18

## Slide 19
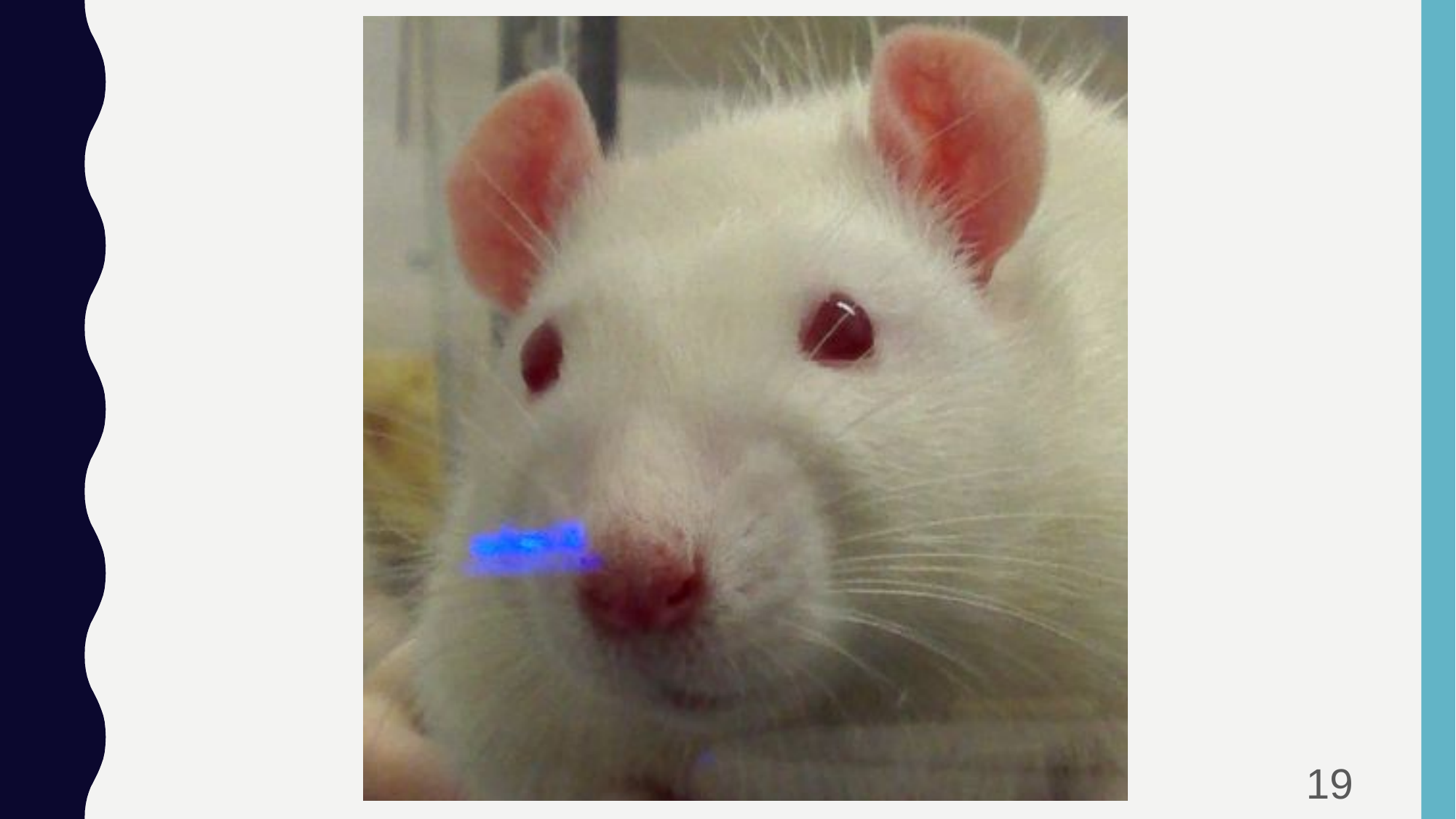

19

## Slide 20
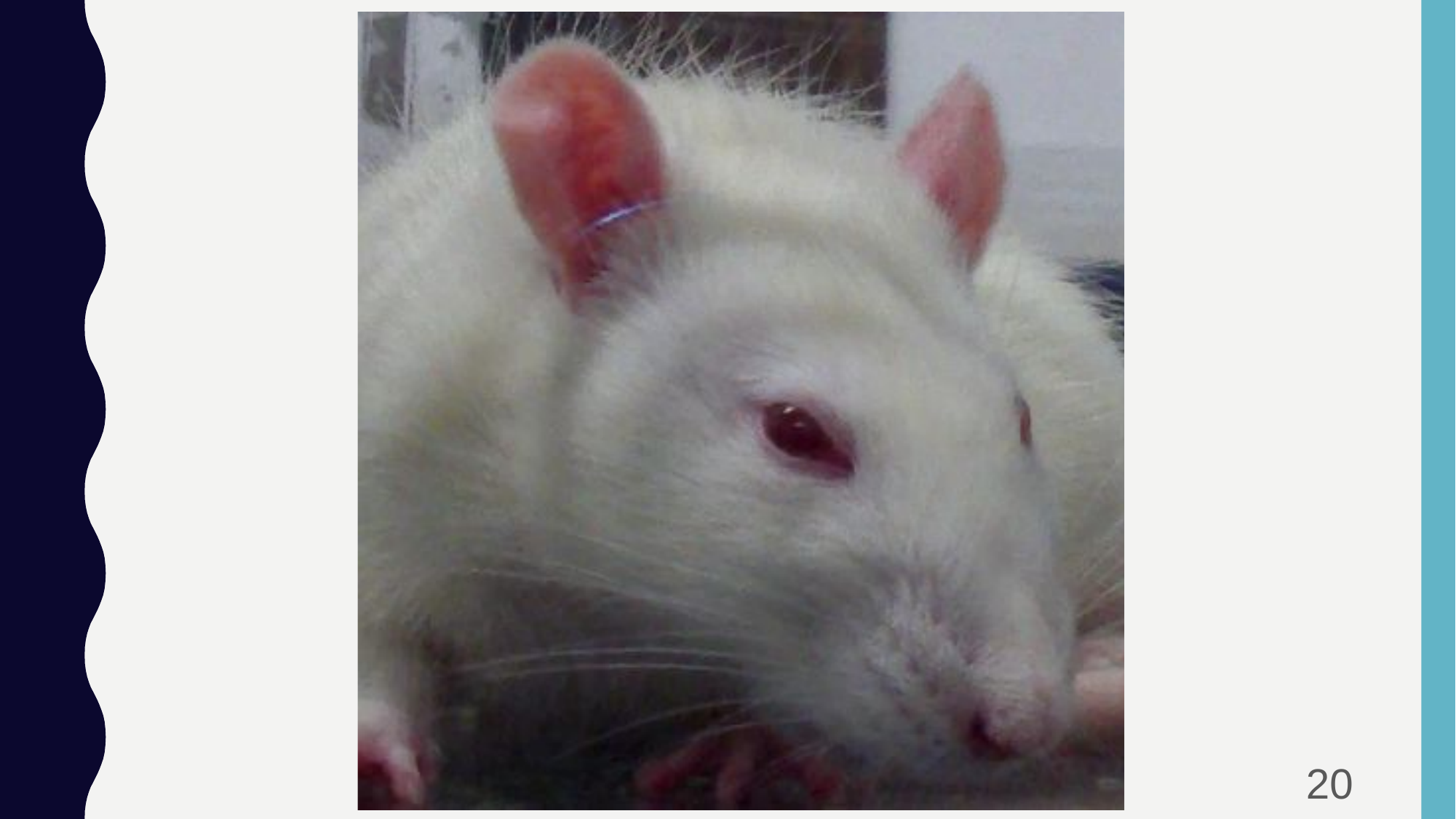

20

## Slide 21
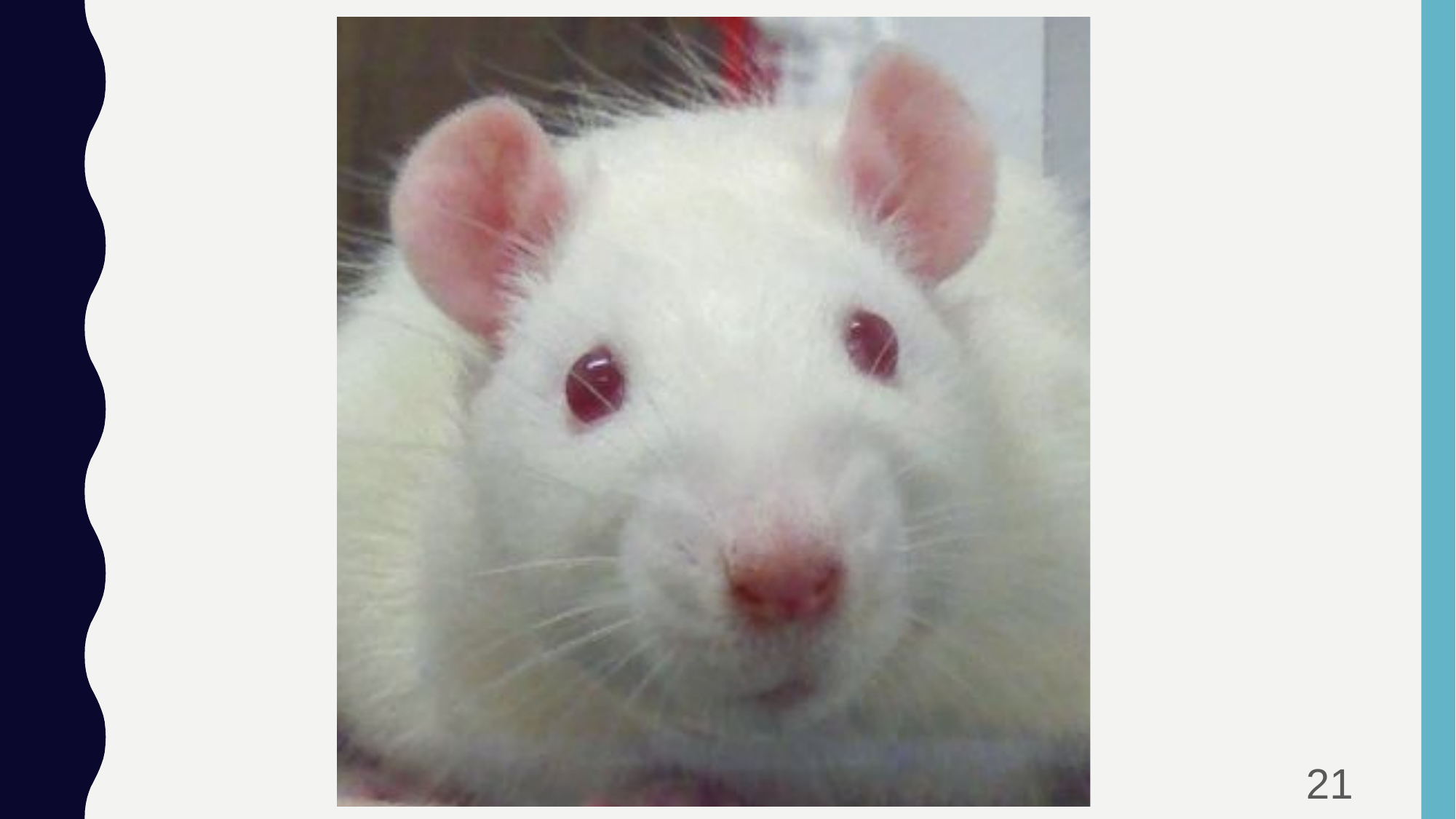

21

## Slide 22
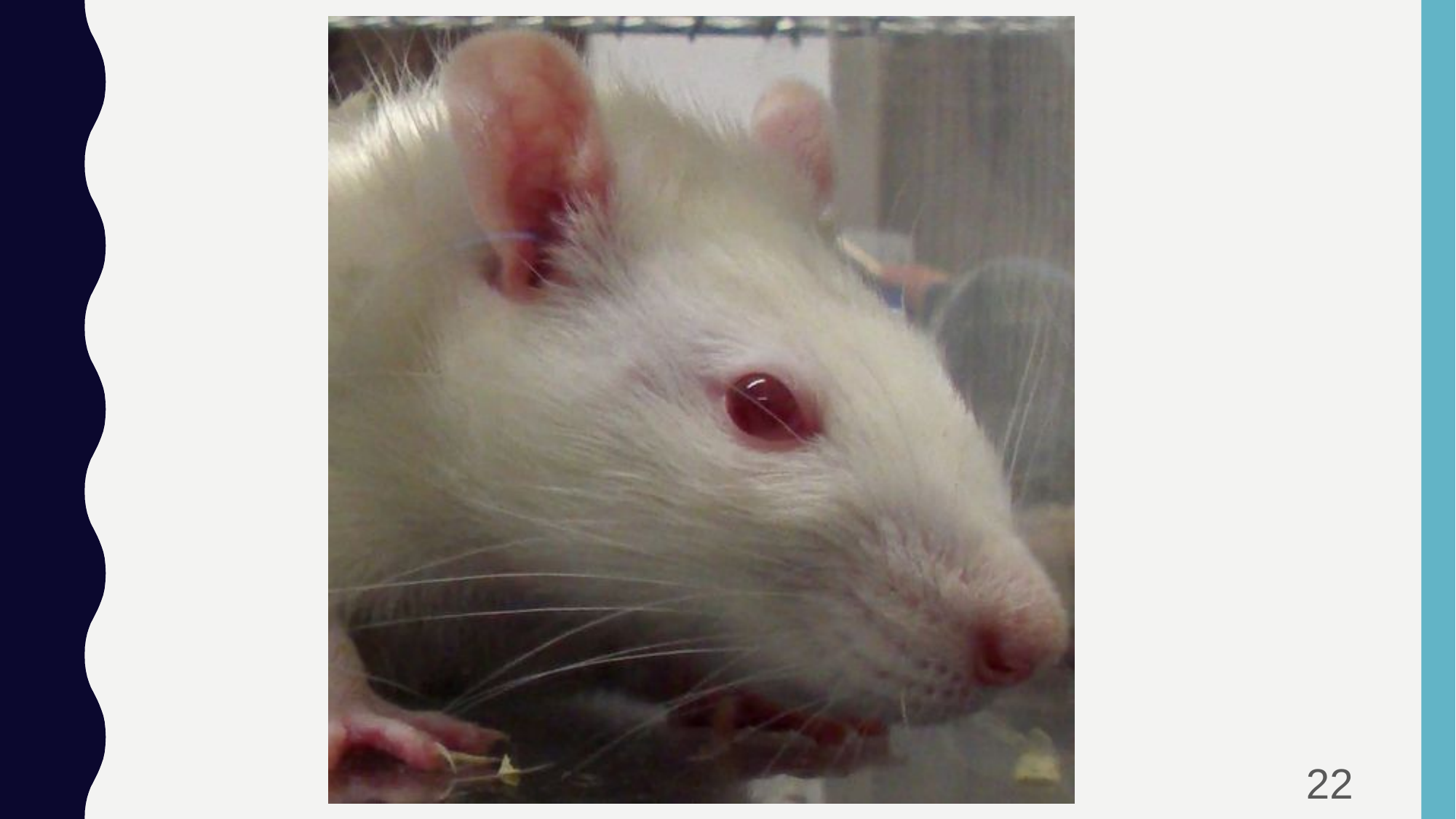

22

## Slide 23
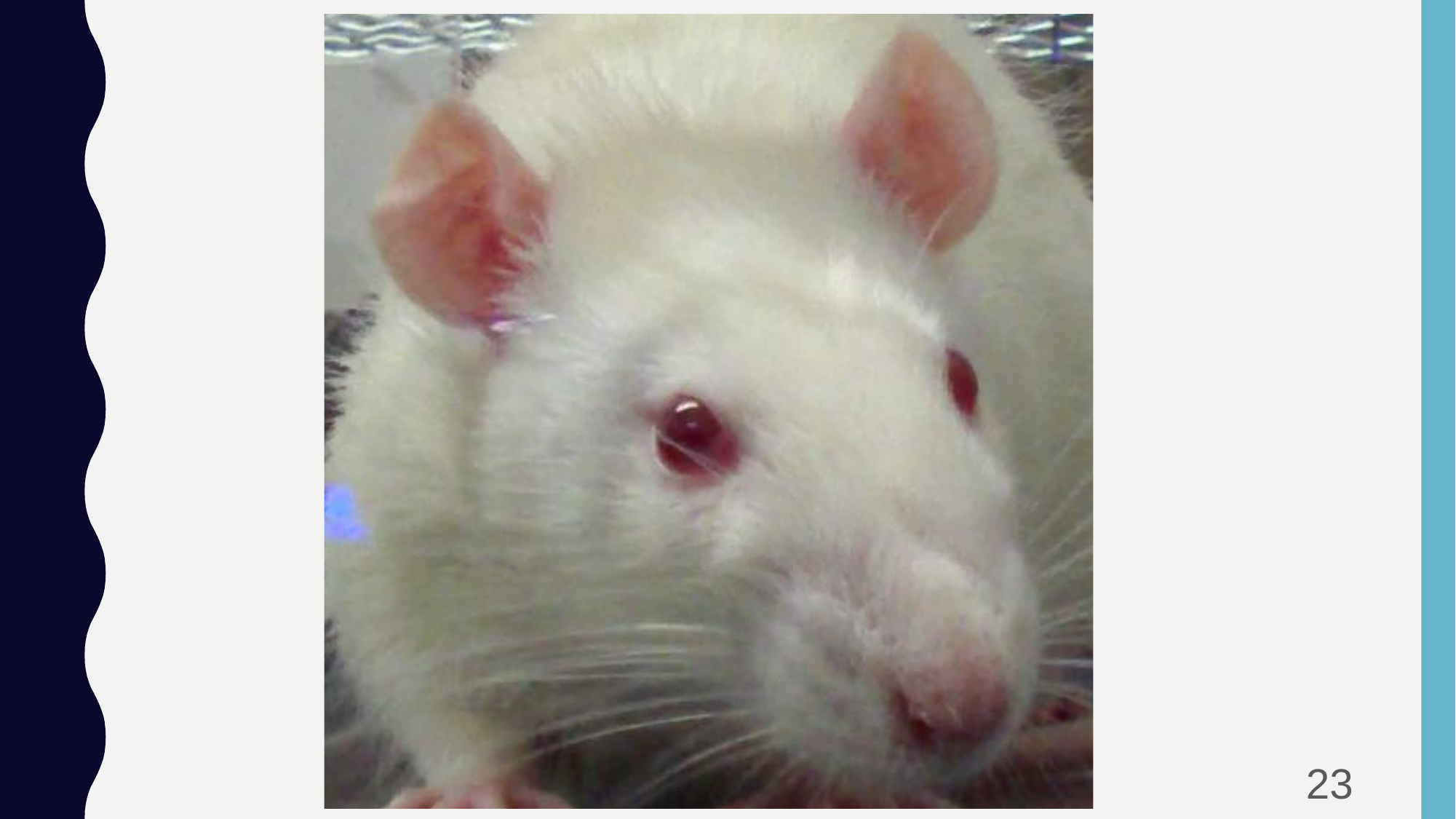

23

## Slide 24
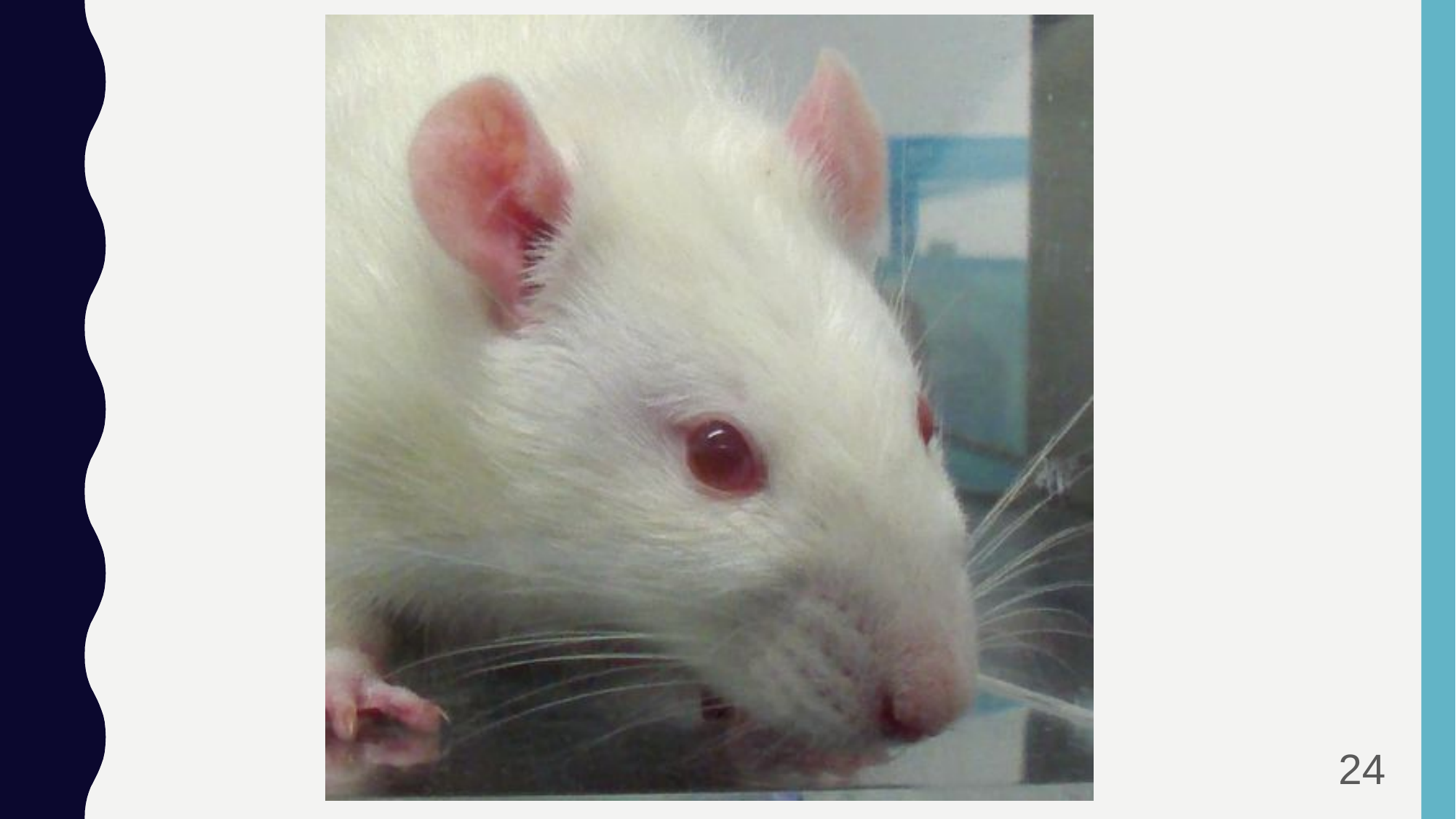

24

## Slide 25
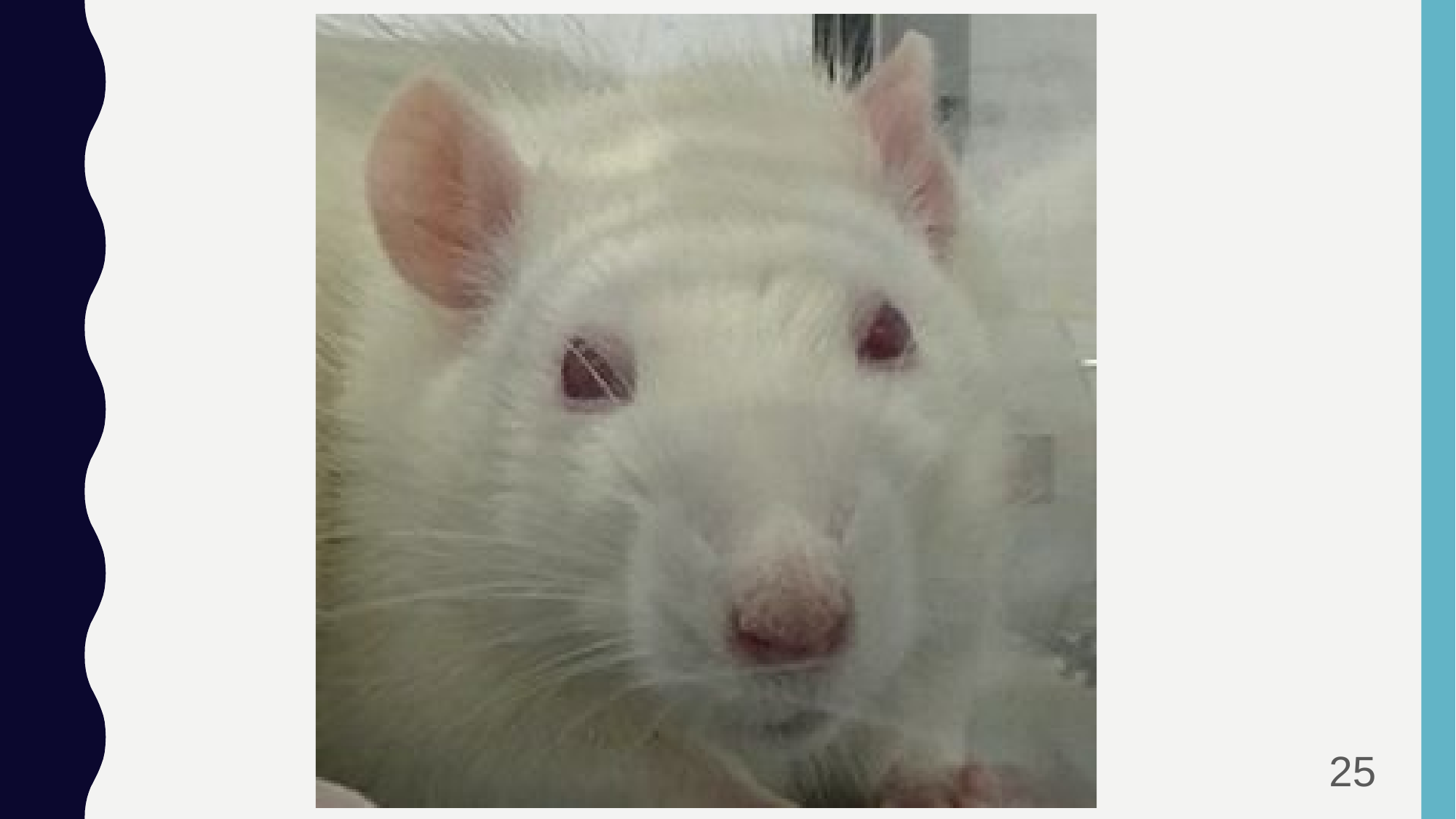

25

## Slide 26
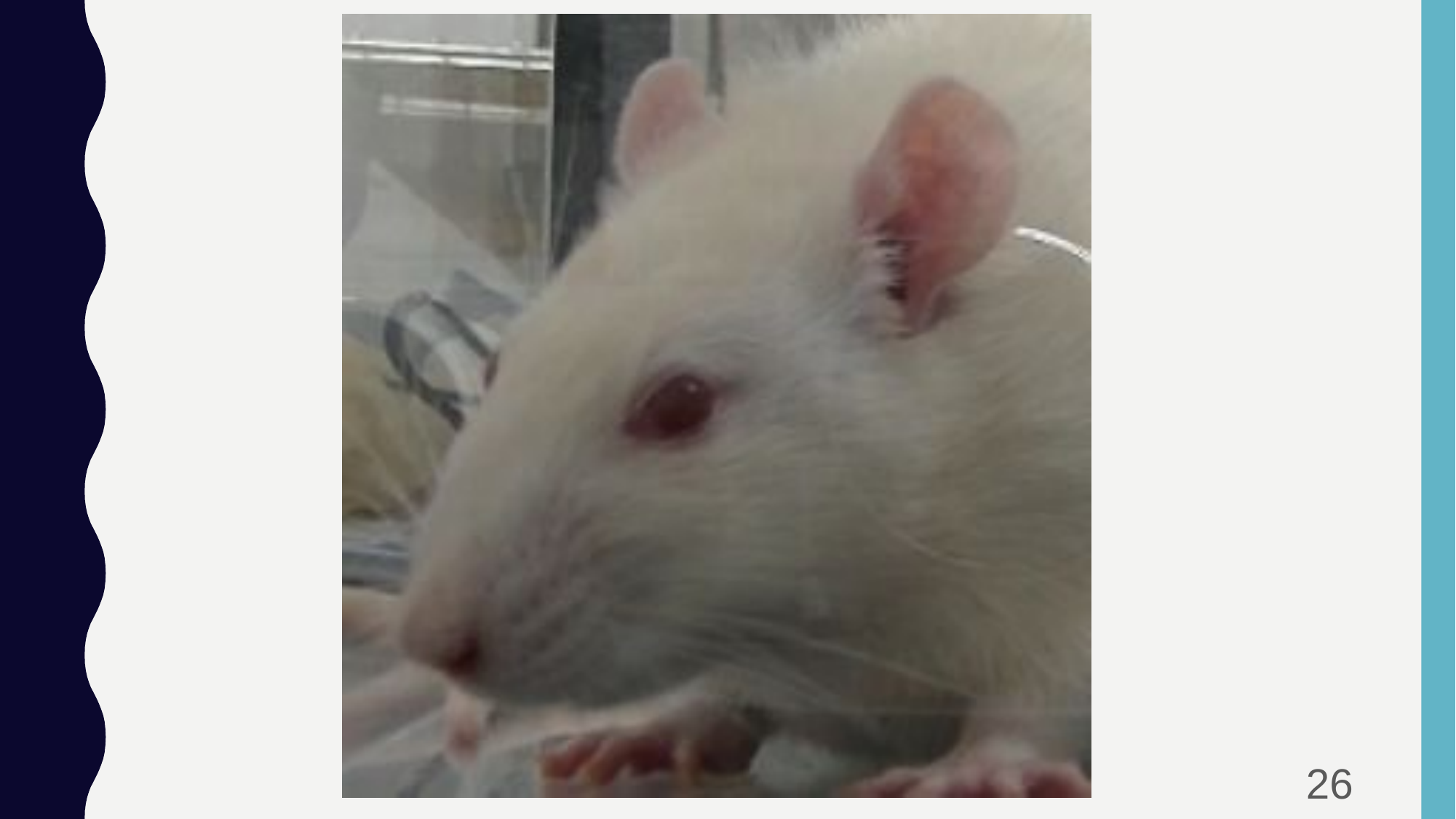

26

## Slide 27
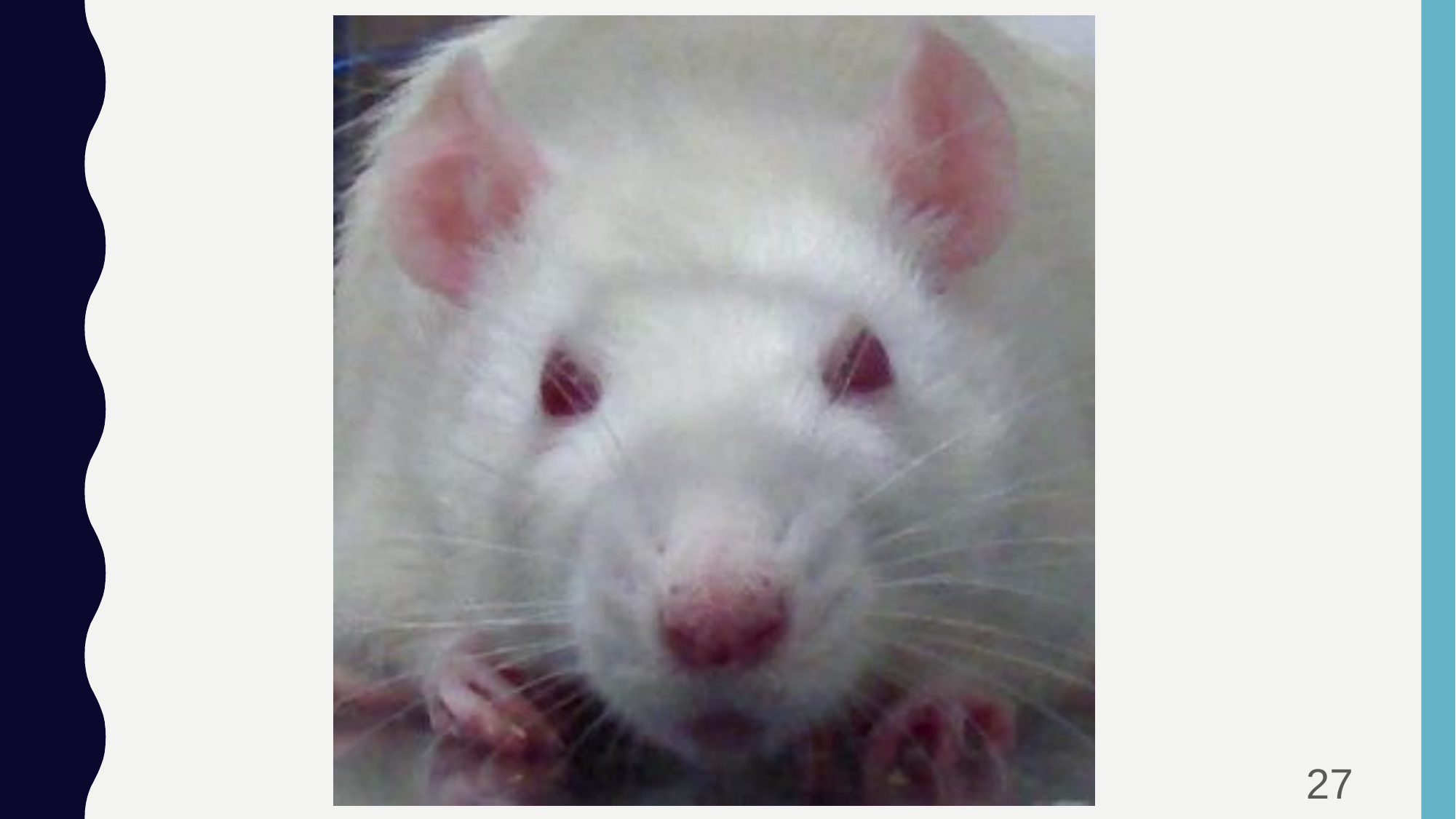

27

## Slide 28
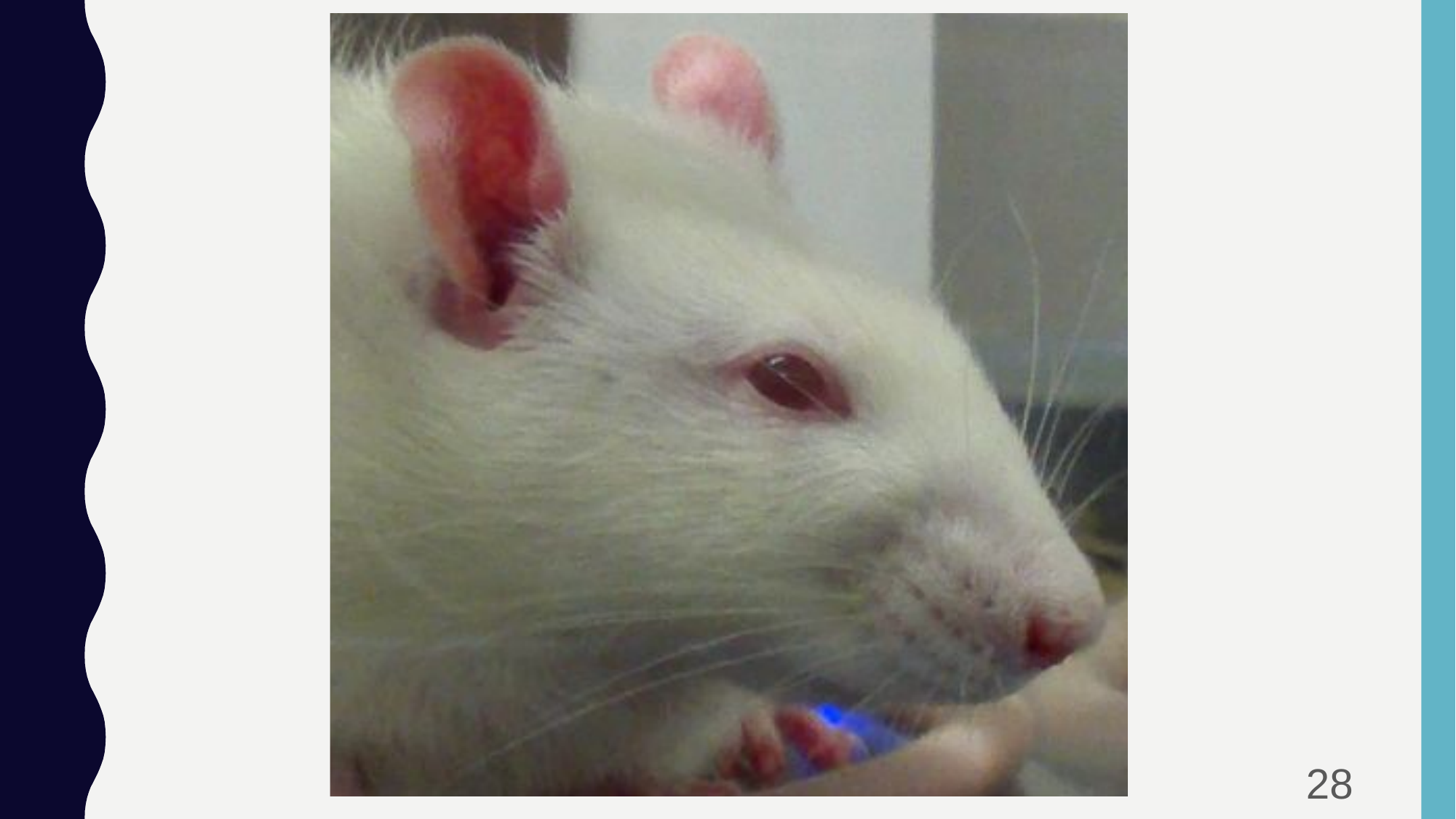

28

## Slide 29
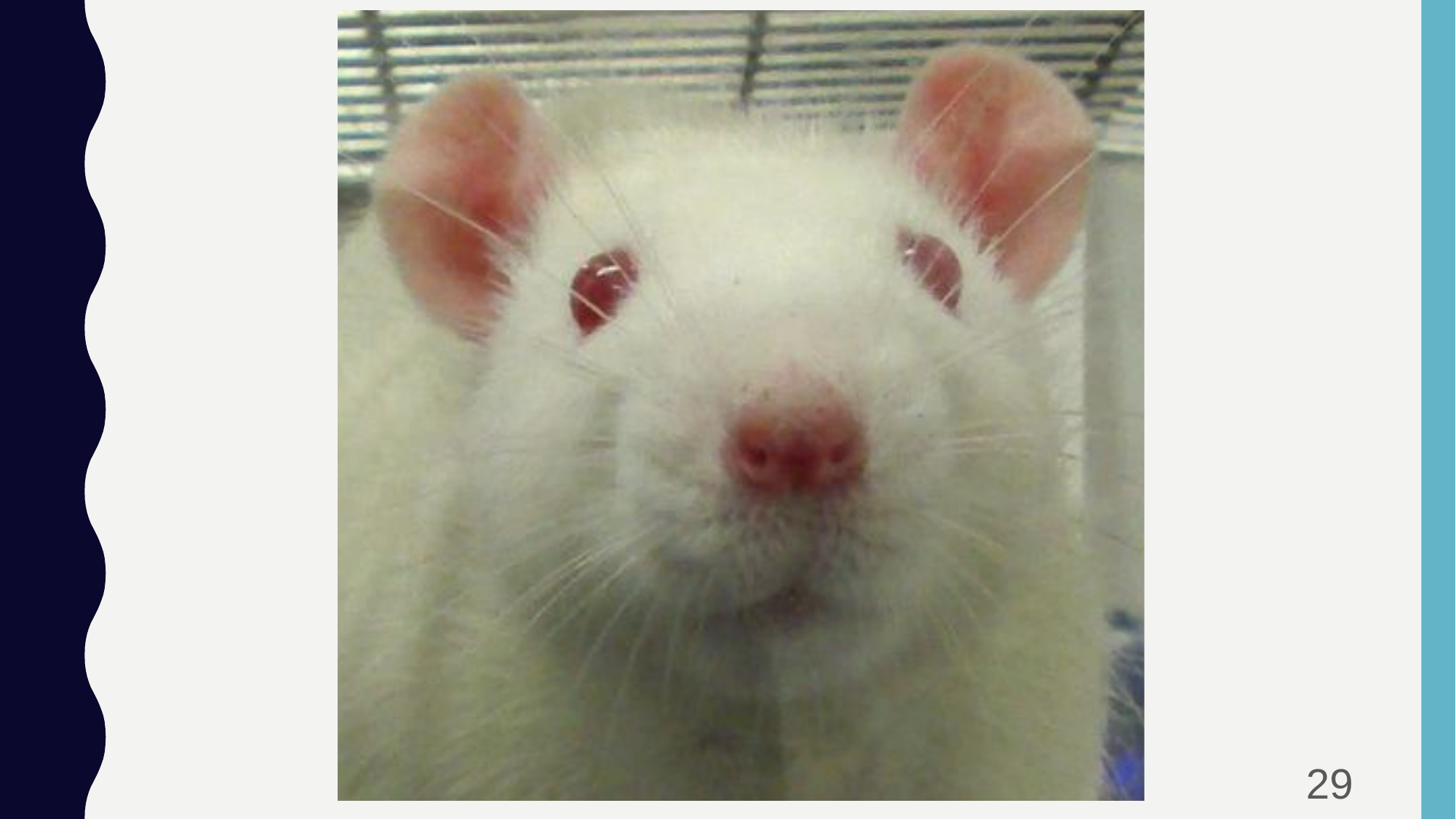

29

## Slide 30
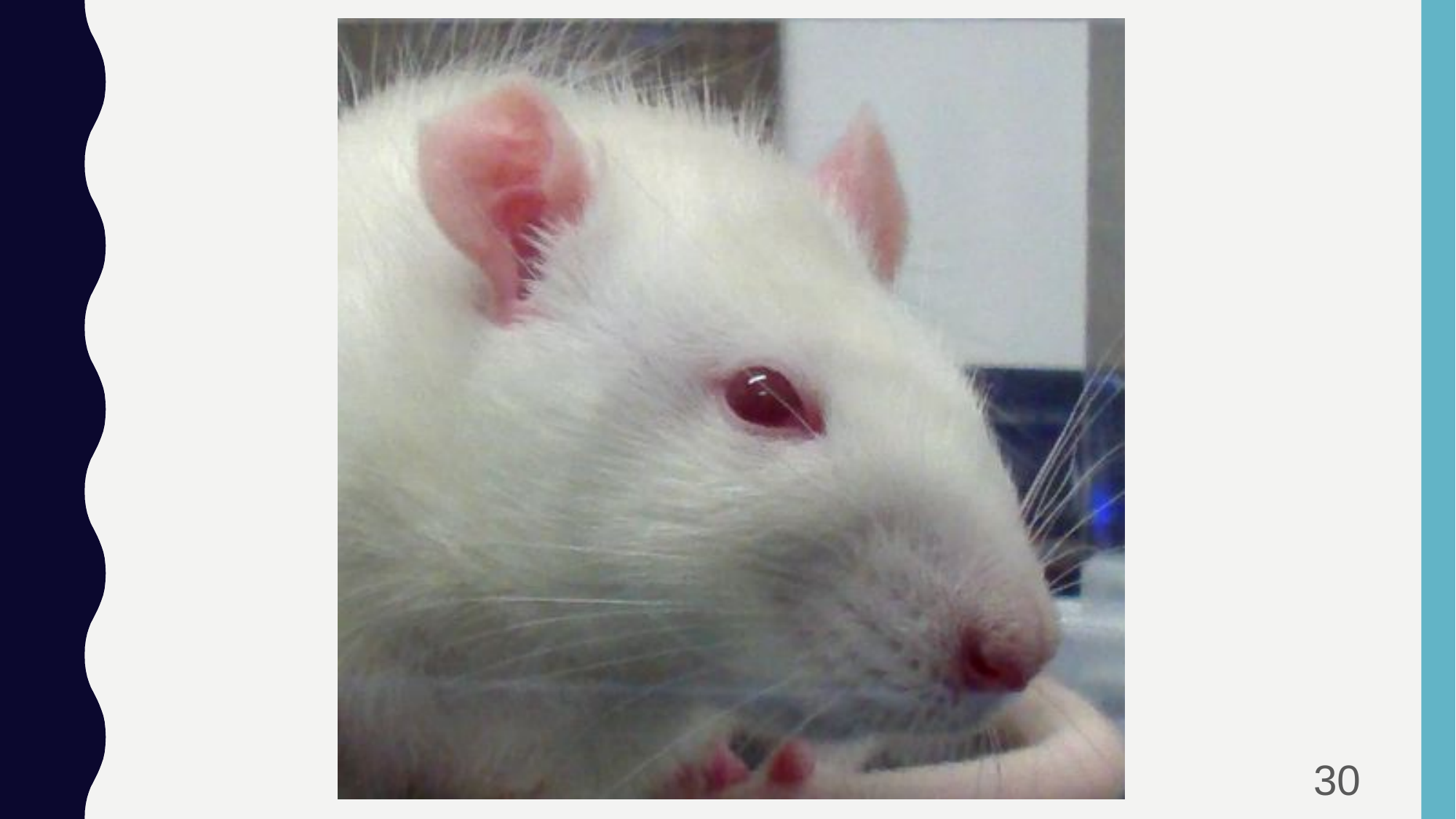

30
